# Estimating distribution and abundance of wide‐ranging species with integrated spatial models: Opportunities revealed by the first wolf assessment in south‐central Italy

**DOI:** 10.1002/ece3.11285

**Published:** 2024-05-13

**Authors:** Vincenzo Gervasi, Paola Aragno, Valeria Salvatori, Romolo Caniglia, Daniele De Angelis, Elena Fabbri, Valentina La Morgia, Francesca Marucco, Edoardo Velli, Piero Genovesi

**Affiliations:** ^1^ Istituto Superiore per la Protezione e la Ricerca Ambientale Roma Italy; ^2^ Federparchi—Italian Federation of Parks and Natural Reserves Roma Italy; ^3^ Istituto di Ecologia Applicata Roma Italy; ^4^ Department for the Monitoring and Protection of the Environment and for Biodiversity Conservation, Unit for Conservation Genetics (BIO‐CGE) Istituto Superiore per la Protezione e la Ricerca Ambientale Ozzano dell'Emilia Italy; ^5^ Department of Life Sciences and Systems Biology University of Torino Torino Italy

**Keywords:** genetic sampling, large carnivores, monitoring, population modelling, spatial capture‐recapture, transboundary wildlife

## Abstract

Estimating demographic parameters for wide‐ranging and elusive species living at low density is challenging, especially at the scale of an entire country. To produce wolf distribution and abundance estimates for the whole south‐central portion of the Italian wolf population, we developed an integrated spatial model, based on the data collected during a 7‐month sampling campaign in 2020–2021. Data collection comprised an extensive survey of wolf presence signs, and an intensive survey in 13 sampling areas, aimed at collecting non‐invasive genetic samples (NGS). The model comprised (i) a single‐season, multiple data‐source, multi‐event occupancy model and (ii) a spatially explicit capture‐recapture model. The information about species' absence was used to inform local density estimates. We also performed a simulation‐based assessment, to estimate the best conditions for optimizing sub‐sampling and population modelling in the future. The integrated spatial model estimated that 74.2% of the study area in south‐central Italy (95% CIs = 70.5% to 77.9%) was occupied by wolves, for a total extent of the wolf distribution of 108,534 km^2^ (95% CIs = 103,200 to 114,000). The estimate of total population size for the Apennine wolf population was of 2557 individuals (SD = 171.5; 95% CIs = 2127 to 2844). Simulations suggested that the integrated spatial model was associated with an average tendency to slightly underestimate population size. Also, the main contribution of the integrated approach was to increase precision in the abundance estimates, whereas it did not affect accuracy significantly. In the future, the area subject to NGS should be increased to at least 30%, while at least a similar proportion should be sampled for presence‐absence data, to further improve the accuracy of population size estimates and avoid the risk of underestimation. This approach could be applied to other wide‐ranging species and in other geographical areas, but specific a priori evaluations of model requirements and expected performance should be made.

## Graphical abstract

To produce wolf distribution and abundance estimates for the whole south‐central portion of the Italian wolf population, we developed an integrated spatial model, based on an occupancy and a spatially explicit capture‐recapture model. The estimate of total population size for the Apennine wolf population was of 2557 individuals (SD = 171.5; 95% CIs = 2127 to 2844). This approach could be applied to other wide‐ranging species and in other geographical areas, but specific a priori evaluations of model requirements and expected performance should be made.

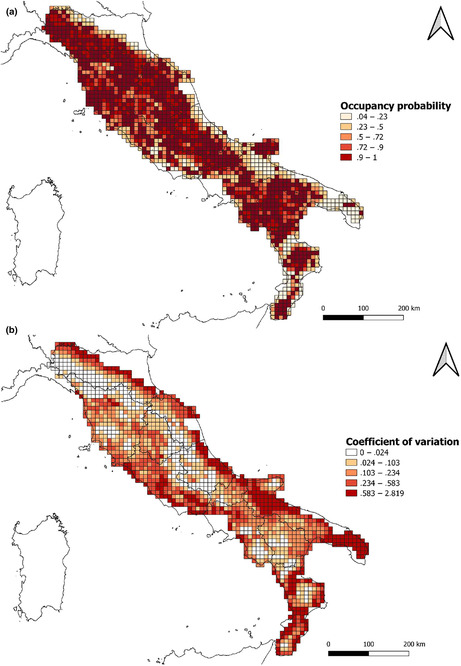

## 1 INTRODUCTION

In recent years, the concurrent emergence of new investigation technologies and advanced statistical tools has greatly improved the ability of ecologists to monitor animal populations in space and time, providing an insight which would have been unachievable just a few decades ago (Allan et al., 2018). Nowadays, the use of Global Positioning System (GPS) collars, drones, photo traps, non‐invasive genetic sampling (NGS), environmental DNA, etc., allows the collection of large datasets at both the population and individual levels (Beng & Corlett, 2020; Oliver et al., 2023; Schad & Fischer, 2022). These data are routinely used to produce distribution and abundance estimates and to inform population management and conservation. On the other hand, new analytical tools, such as occupancy models and spatially explicit capture‐recapture models (SCR), have become standard methods for the demographic monitoring of animal populations. These methodologies allow the consideration of imperfect detection, but also individual and spatial variation in detection and movement rates, thus increasing the robustness of the estimated demographic parameters (Mackenzie et al., 2002; Tourani, 2022).

Despite the technological and methodological advancements, though, estimating basic demographic parameters for wide‐ranging and elusive species living at low density remains a challenging exercise, especially when there is a need to estimate these parameters at the scale of an entire country or even at a transboundary level. Large carnivores and marine mammals are among the most typical examples of such a challenge. They live at very low densities, each individual in the population can potentially move over very large distances, and their populations are bound to occupy wide geographical areas to be sustained, spanning across one or several countries (McDonald, 2004). Therefore, monitoring their populations requires a massive sampling effort, which is often beyond reach for national agencies. The estimation of population size for brown bears (*Ursus arctos*), lynx (*Lynx lynx*), wolverines (*Gulo gulo*), and wolves (*Canis lupus*) in Norway and Sweden is one of the few examples of a systematic large‐scale (>500,000 km^2^) NGS program on several wide‐ranging, elusive species (Bischof, Dupont, et al., 2020; Bischof, Milleret, et al., 2020). Similarly, Lauret et al. (2023) estimated the abundance and density of bottlenose dolphins (*Tursiops truncatus*) in a vast portion of the North‐Western Mediterranean Sea (>200,000 km^2^), using a combination of boat surveys and aerial line transects. For most of the populations of these and similar species, though, the establishment of a spatially exhaustive and systematic national monitoring program remains a challenge. The tools and techniques are available, but the effort required is often too big. As a result, several population monitoring programs make use of a mix of different opportunistic data sources or restrict their effort to portions of the whole population (López‐Bao et al., 2018; Popescu et al., 2017). Often, in fact, a combination of individual recognition data, presence‐absence data, dead‐recoveries, etc., is collected opportunistically or even incidentally by field technicians, hunters, or citizens (Cretois et al., 2020; Ražen et al., 2020). While being of value locally, these data provide information only about local abundance, a minimum number of individuals present in the population, minimum distribution maps, etc., being of little support for large‐scale management and conservation.

The last decade, though, has seen an increasing number of applications, in which individual recognition data and presence‐absence data were integrated to enhance the performance of spatial models. Kéry and Royle (2016) showed that both data types can be used to inform a SCR model, in particular through a link between the location of individual activity centres and the probability to collect presence‐absence data at a given sampling site. Therefore, the recent availability of integrated spatial models offers a promising opportunity to make the most out of these different data types (Chandler & Clark, 2014; Schaub & Abadi, 2011). Integrated spatial models allow to combine and simultaneously analyse data deriving from different underlying sampling processes, thus improving the accuracy, precision, and robustness of demographic parameter estimates (Blanc et al., 2014; Chandler & Clark, 2014).

Wolves in south‐central Italy are a typical example of a wide‐ranging species, distributed over large portions of land, with a lack of ecological knowledge and some limitations in the applicability of a comprehensive sampling design. Wolves in Italy were almost extinct by the 1970s, surviving only in a few isolated nuclei in south‐central Apennines (Zimen & Boitani, 1975). Since then, and in line with the general recovery of large carnivores across all Europe (Chapron et al., 2014), the species has progressively recovered its former range, recolonized the Alps, and eventually expanded into France, Switzerland, and Austria (Valière et al., 2003). Although the Italian wolf population is now continuously distributed across the whole country, from a management point of view two interconnected populations are identified (Fabbri et al., 2007), one in the Alps and the other in the remaining south‐central portion of the peninsula, mainly along the Apennine Mountains chain. Both in the areas of historical presence and in those of recent comeback, wolf presence has varying levels of impact on livestock farming and social conflicts in areas of their historical range and recent recolonization, requiring management and conflict mitigation (Gervasi et al., 2021). Despite these management needs, a formal population size estimate at the national level, and for its south‐central portion, is currently lacking, although preliminary attempts have been made using a collection of local studies (Galaverni et al., 2016).

**FIGURE 1:**
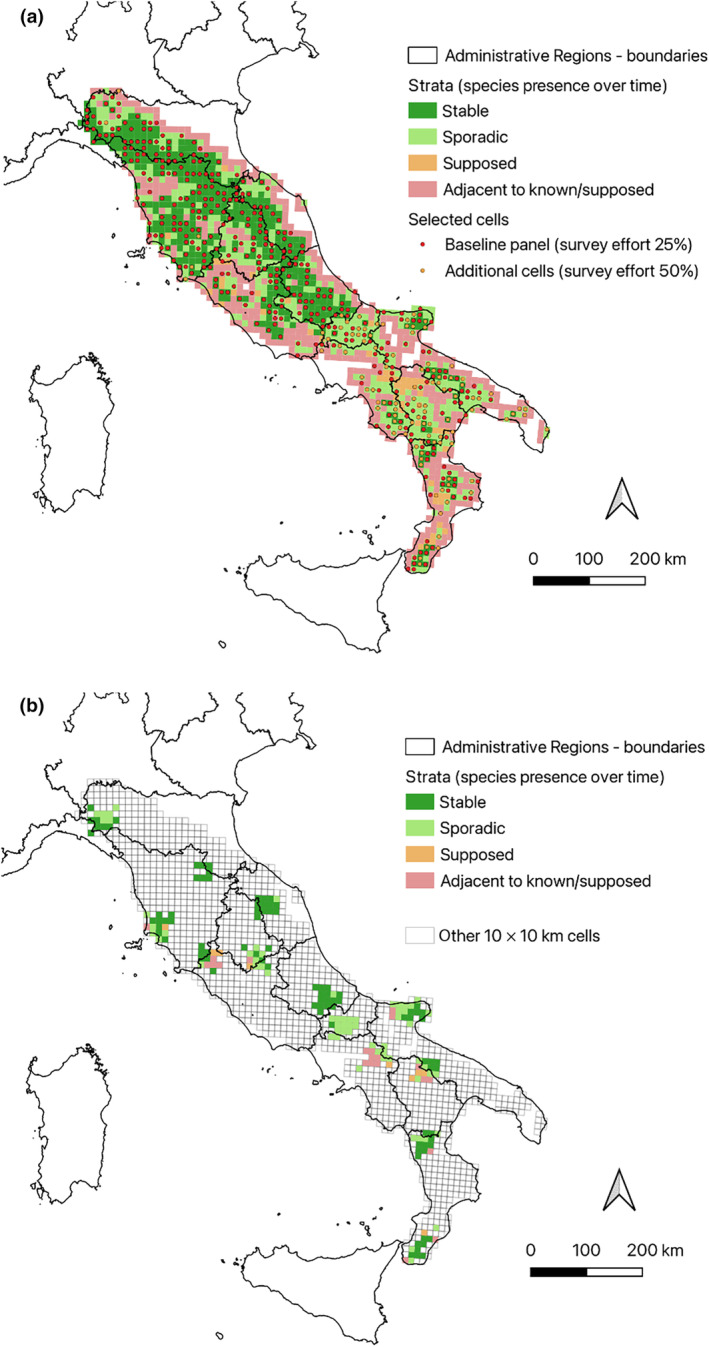
(a) Stratification of the study area and selected sampling units for the extensive survey in south‐central Italy. (b) Cells selected for the intensive survey.

In 2020, a national population estimation project was launched, as a result of a simultaneous and standardized sampling of the two portions of the populations. For the alpine portion, the project resulted in a SCR‐based density and abundance estimate, which allowed to define key population metrics with direct relevance for conservation and management (Marucco et al., 2023). A different approach was adopted in south‐central Italy, owing to the vast area supposedly occupied by the species (about 150,000 km^2^; see Figure 1), the lack of previous knowledge in large portions of the wolf distribution range, and the limitations in the maximum achievable field effort. The field design was based on a stratified random sampling, aimed to collect both presence signs and NGS data for a portion of the whole population. In this paper, we describe how we developed an integrated spatial model, combining an occupancy model and an SCR model, to produce wolf distribution and abundance estimates for the south‐central portion of the Italian wolf population, making the most out of the data collected during a 7‐month sampling campaign.

To assess the expected performance of our sampling and analytical design, and to produce guidelines for future applications, we also performed a simulation‐based assessment, which allowed us to estimate the best conditions for optimizing sub‐sampling and integrated spatial modelling, in order to produce accurate and precise enough estimates of population size. We discuss the opportunities and risks of such an approach, which is potentially suitable for several monitoring programs of large carnivores and other wide‐ranging species, but also requires caution and a proper a priori evaluation before its application.

## 2 MATERIALS AND METHODS

### 2.1 Sampling design

To estimate wolf abundance and distribution in south‐central Italy, we designed sampling by overlaying the 10 × 10 km grid adopted at the European level for the Habitats Directive 92/43/EEC reporting (https://www.eea.europa.eu/data‐and‐maps/data/eea‐reference‐grids‐2) to the 11 administrative Regions overlapping the study area (Figure 1a). Given the large size of the area and the logistic constraints, we randomly subsampled the cells to be surveyed, based on a stratified design. We distributed sampling effort according to the variability of the parameter of interest, that is the supposed local wolf density (McDonald, 2004). For this purpose, we considered that the best available proxy for wolf density was the opportunistic information on the species' presence over time. Therefore, we classified all grid cells into three strata of stable, sporadic, and supposed presence, based on heterogeneous and asynchronous previous data (e.g. reporting under the Habitats Directive). Additionally, we defined a fourth stratum, including all the cells adjacent to those of known or possible presence, to correct for some gaps in the available information on species presence (Figure 1a).

The data collection strategy was articulated into (i) an extensive survey aimed at collecting wolf presence data; (ii) an intensive survey carried out in 11 sampling areas, defined by a 3 × 3 arrangement of 9 cells, and aimed at collecting non‐invasive genetic samples (Figure 1b).

For the extensive survey, we ensured the selection of spatially balanced and representative sample of cells via a Generalized Random Tessellation Stratified (GRTS) design (Stevens & Olsen, 2004). We defined the inclusion probability of each grid cell in the final sample as a function of its stratum, allocating a proportionally larger number of cells in the strata where more variance in the parameter of interest was expected. We simultaneously extracted 25% of the cells across the four strata. Because the information about the species' presence was generally scarcer in the southern part of the study area, we oversampled to 50% in the five southernmost administrative regions, thus compensating for the imbalance in the baseline information and for the expected larger variance in local density. The application of the sampling algorithm resulted in the probabilistic selection of 449 grid cells (254, 153, and 8 grid cells, respectively, in the stable, sporadic, and supposed presence strata, and 34 in the cells adjacent to those of known/supposed presence; Figure 1a).

To identify the 11 intensive survey areas, we adopted a design which involved both a random and a systematic component. We constrained the stratified random selection to the boundaries of the administrative regions, with the additional criterion that each region should include one intensive area. To this aim, we first selected the central 10 × 10 km grid cell of each intensive area in each region, then extended it to the eight nearest‐neighbouring cells, provided they fell within the strata defined above. Based on the first data gathered through the extensive survey, we identified two additional intensive areas in the northern and southernmost parts of the study area, bringing the total number to 13. The intensive data collection was finally carried out in 186 10 × 10 km cells (Figure 1b).

### 2.2 Data collection

Both the intensive and extensive data collection took place from 1st October 2020 to 30th April 2021. We considered this period as the most promising for detecting the species, as it fell between the main dispersal events of spring and fall (Mech & Boitani, 2019). Also, because new wolf pups typically are born at the end of spring, we most likely did not include the recruitment peak in the sampling period and could assume that the population remained demographically closed throughout the data collection period (Dupont et al., 2019). A network of field staff from several institutions concerned with wildlife management (*N* = 344), volunteers (*N* = 431) and forestry service staff (*N* = 725) systematically collected field data on a network of transects, selected along roads and trails, and through photo traps placed across the study area. Field workers were trained through 106 training courses and 11 repetitions of a course in e‐learning mode. Data collection occurred under the supervision of 20 highly experienced technicians, who inspected the reliability of all presence signs before updating the database monthly. In the intensive areas, we collected both wolf presence sign data and non‐invasive genetic samples, whereas in most of the extensive areas we only collected presence signs. We applied the SCALP criteria to categorize each wolf presence sign into C1, C2, or C3 (Molinari‐Jobin et al., 2012), representing increasing levels of uncertainty in species attribution. Targeted wolf presence signs included scats, photos, tracks, sightings, urine, hairs, predation sites, howling, and wolf carcasses. Targeted non‐invasive genetic samples included fresh scats, blood, saliva, and muscular tissues retrieved from wolf carcasses. In the intensive areas, we collected faecal samples using cotton swabs, whereas in the extensive areas we collected and stored a small portion of the scat in tubes containing 96% ethanol (Velli et al., 2019). Typically, we visited transects monthly in the intensive areas, and bi‐monthly in the extensive areas. In addition to the data collected through systematic sampling, we also considered additional wolf data retrieved opportunistically, that is verified data occasionally collected during field activities unrelated to the sampling campaign.

### 2.3 Molecular analyses

#### 2.3.1 DNA extraction and amplification

DNA was automatically extracted from 1600 non‐invasively collected samples (1592 faecal swabs, 3 hairs, 3 urines and 2 salivary swabs; Velli et al., 2019) and 32 invasive biological materials (30 muscular tissues from found dead individuals and 2 blood samples from rescued wolves) using a QIAcube HT® robotic workstation and the Qiagen DNeasy Blood & Tissue Kit, following the manufacturer's instructions.

Each DNA sample was amplified by Polymerase Chain Reaction (PCR) and genotyped, through a multiple‐tube approach (Caniglia et al., 2014), at 12 unlinked autosomal canine microsatellite (STR) *loci* (Table S1.) already successfully applied for individual identifications in long‐term non‐invasive monitoring projects about population dynamics of the Italian wolf (Caniglia et al., 2014; Fabbri et al., 2018), for forensic applications (Caniglia et al., 2016; Velli et al., 2022) and for the reliable discrimination between wolves, dogs and their first two‐three generation hybrids through Bayesian assignment procedures (Caniglia et al., 2020). DNA samples were also genotyped through a multiple‐tube approach, at (i) a portion of the amelogenin gene, to molecularly determine their gender, (ii) at 4 Y‐chromosome STRs (Table S1.) to determine paternal haplotypes in male individuals, and (iii) at a dominant 3‐bp deletion at the *β*‐defensin CBD103 gene (the K‐*locus*) coding for the black coat colour in canids (Caniglia et al., 2013).

The 4–8 replicated amplifications per *locus* per sample foreseen by the multi‐tube approach were used to estimate sample reliability by the software RELIOTYPE (Miller et al., 2002), reconstruct consensus genotypes, and assess the occurrence of allelic dropouts and false alleles by the software GIMLET V.1.3.3 (Valière, 2002). GIMLET was also used to identify identical genotypes and individual recaptures. Unique genotypes were also typed at 250 bp of the mitochondrial DNA control region (mtDNA CR) containing diagnostic mutations (Supporting information) to distinguish Italian wolves from European wolves and dogs (Caniglia et al., 2013).

The software GENALEX (Peakall & Smouse, 2012) was used to estimate the mean number of different (*N*_A_) and effective (*N*_E_) alleles, the probability of identity (*p*_ID_) and the expected *p*_ID_ among full‐sib dyads (*p*_IDsibs_), and values of observed (H_O_) and expected (H_E_) heterozygosity.

Extraction, amplification, and post‐amplification procedures of both non‐invasive and muscular DNA were carried out in separate rooms reserved to low‐ and medium‐template DNA samples, adding a blank control (no biological material) during DNA extraction, and a blank (no DNA) and a positive (good quality and known wolf‐DNA profile) controls during DNA amplification to check for possible contaminations (for details see the Supporting Information).

#### 2.3.2 *Taxon* identification

The 12‐STR *multilocus* genotypes were assigned to their *taxon* of origin (wolf, dog or admixed), independently of any a priori non‐genetic information, through a Bayesian clustering procedure implemented in the program PARALLEL STRUCTURE (Besnier & Glover, 2013), an R package implementing STRUCTURE, following indications reported in Caniglia et al. (2020). Assignments were integrated with the information derived from the uniparental (mtDNA CR, four Y‐linked STRs) and coding (K‐*locus*) markers, which were used to confirm the *taxon* identification or, in case of admixed individuals, to provide the directionality of the hybridization (Caniglia et al., 2020).

### 2.4 Statistical modelling

#### 2.4.1 Occupancy model

For the estimation of wolf distribution, we built and analysed a single‐season, multiple data‐source, multi‐event occupancy model (Mackenzie et al., 2002; Miller et al., 2011; Pacifici et al., 2017), which accounted for the different types of sampling processes involved in data collection (transects and photo traps) and for the possibility of species misidentification when collecting wolf scats in areas where also dogs were present.

We divided the sampling period into four sessions, three of them comprising 2 months (October–November, December–January, February–March), whereas the last one only included the data collected in April. We then built two sampling matrices, one for the presence data derived from photo traps, one for the data collected along transects, and initially coded the matrices with a simple binary code (1 = species detected; 0 = species not detected). For the wolf presence data derived from transects, though, we could not exclude the possibility that dog scats were erroneously attributed to wolves, thus generating a false detection in the dataset. To account for this risk, we identified three different types of wolf detections along transects and explicitly included the misidentification probability into the model. This was possible because a portion of the wolf samples collected along transects were genetically analysed and identified as belonging to dogs, thus providing a basis for the estimation of the misidentification probability. The three detection types were based on the use of two classification criteria, one derived from field work, the other from the genetic lab results, and allowed to distinguish samples that were certainly belonging to wolves, samples certainly belonging to dogs and uncertain samples. A fourth type of event was assigned to cells with no detection (see Table 1). Then, based on two possible occupancy states (occupied by wolves, not occupied by wolves) and four types of detection events, we built a 2 × 4 detection matrix for the data collected along transects.

$$\left[\begin{array}{ccccc} & \mathrm{No}\;\text{detection}\;\left(0\right) & \text{Wolf}\;\left(1\right) & \mathrm{Dog}\;\left(2\right) & \text{Unknown}\;\left(3\right)\\ \text{Not occupied}\;\left(0\right) & \left(1-P_{1,x,t}\right) & 0 & P_{1,x,t}*G_{x} & P_{1,x,t}*\left(1-G_{x}\right)\\ \text{Occupied}\;\left(1\right) & \left(1-P_{2,x,t}\right) & P_{2,x,t}*G_{x}*\left(1-M_{x}\right) & P_{2,x,t}*G_{x}*M_{x} & P_{2,x,t}*\left(1-G_{x}\right) \end{array}\right]$$

**TABLE 1:** Description of the criteria used to classify each scat‐based wolf detection into four detection types, to account for a possible species misidentification in the occupancy model. The data were collected along transect in south‐central Italy between October 2020 and April 2021.

Index	Scat collected	Field classification	Scat analysed genetically	Genetic lab results	
0	No	—	—	—	Possible species absence
1	Yes	Wolf	Yes	Wolf	Wolf presence ascertained
2	Yes	Wolf	Yes	Dog	Misidentification error
3	Yes	Wolf	No	—	Possible species presence

In the detection matrix, *P*_*1,x,t*_ was the probability to detect a dog sign in the unoccupied cell *x* during session *t*, *P*_*2,x,t*_ was the probability to detect a presence sign (dog or wolf) in the occupied cell × during session *t*, *G*_*x*_ was the probability for a sample collected in cell *x* to be genetically analysed in the lab, and *M*_*x*_ was the probability that a sample classified as wolf in the field was later classified as belonging to a dog in the lab. Parameter *G* was just a dummy variable, which was fixed to one for the intensive cells, whose samples were genetically analysed, and fixed to zero for the cells not included in the genetic study. Based on this parameterization, *P*_*1*_ represented the probability of a false detection in the occupancy model.

For the data derived from photo traps, we assumed that all wolf detections were correct, as they were based on the visual inspection of photos by expert technicians. The resulting detection matrix, therefore, only included two underlying states (the same ones used for the previous matrix) and two possible detection events (0 = no detection; 1 = wolf presence detected). In the matrix, *P*_*3,x,t*_ was the probability of detected wolf presence through photo traps in cell *x* during session *t*.

$$\left[\begin{array}{ccc} & \mathrm{No}\;\text{detection}\;\left(0\right) & \text{Wolf}\;\left(1\right)\\ \text{Not occupied}\;\left(0\right) & 1 & 0\\ \text{Occupied}\;\left(1\right) & \left(1-P_{3,x,t}\right) & \psi_{x}*P_{3,x,t} \end{array}\right]$$

We modelled the spatial and temporal variation in all the involved parameters as a function of a set of covariates.

We assumed that cell‐specific occupancy was a realization of a Bernoulli process with index *ψ*. To estimate the occupancy probability *ψ*, we included the average altitude above sea level in each cell, the proportion of cell area covered by forest, the proportion of urbanized area, the total length of primary and secondary roads, the proportion of agricultural land and of natural agricultural areas, the average human density, the terrain ruggedness index, and the number of wild ungulate species available. We derived this variable from the wild ungulate distribution maps produced by Linnell et al. (2020) at the European level, using hunting statistics, citizen science databases, vehicle collisions, scientific papers, and expert assessments on 17 native and non‐native ungulate species. Six of these species, red deer (*Cervus elaphus*), roe deer (*Capreolus capreolus*), chamois (*Rupicapra pyrenaica*), wild boar (*Sus scrofa*), fallow deer (*Dama dama*), and mouflon (*Ovis ammon*) were distributed in at least a portion of our study area and were used to build the species availability index. We also added an individual random effect, which accounted for the residual differences in occupancy probability among cells. We derived landscape variables from the EU‐DEM v1.1 (Copernicus Land Monitoring Service, EEA) and from the Corine Land Cover (2018, Copernicus Land Monitoring Service 2018, EEA). We extracted road density data from OpenStreetMap (OpenStreetMap contributors 2021), aggregating the motorway trunk, primary, secondary, tertiary, and unclassified road classes. We also added a spatial autocorrelation function through a normally distributed individual random term *ε*_*x*_ for each cell. The random effect had mean equal to zero and variance defined as *σ* ^2^ (*D*−*ϕW*), in which *σ* was the standard deviation, *W* was a binary adjacency matrix (1 = bordering, 0 = not bordering), *D* was the diagonal matrix of *W*, and *ϕ* was an estimated parameter controlling the intensity of the spatial correlation.

For the detection probability parameters, we modelled the spatial and temporal variation as a function of sampling effort, measured as the total number of kilometres walked in each cell during each sampling session (for transect‐based data) and as the number of trap nights for each photo trap. We also estimated a different intercept for each sampling session and tested if detection probability was related to the total amount of snow cover in each cell during the sampling period (reanalysis dataset; ERA5‐Land). We also tried to account for the residual individual heterogeneity in detection probability among cells, using a finite‐mixture approach with two classes of heterogeneity (Pledger, 2005), controlled by an additional regression parameter and by parameter *θ*, which defined the individual probability of each cell to belong to one of the two mixtures. As in most of the applications of occupancy models on large carnivores and other wide‐ranging species (Blanc et al., 2014; Lauret et al., 2021), we could not assume perfect closure of the grid cells during the sampling period, that is a cell could possibly be occupied by the species only during a portion of the year. Such violation would have no effect on the estimates, only if animal movements were random between cells, an assumption which cannot be considered as valid for species whose movement are influenced by their non‐random habitat use patterns (Kendall et al., 2013). In such a situation, the occupancy parameter *ψ* is usually interpreted as a probability of use of a given cell, rather than as a classical species presence (Kendall et al., 2013). Therefore, species detection was conditional on both the probability that the species is available for sampling and the probability that the species is using the grid cell during sampling.

#### 2.4.2 SCR model

For the estimation of wolf abundance, we used the dataset derived from the non‐invasive genetic sampling performed in the 13 intensive sampling areas, which comprised the individual identification of all sampled wolves, their sex, the number and location of all the individual genetic captures. We used these data to build a Spatially Explicit Capture‐Recapture model (SCR). To this aim, we used the 10 × 10 km sampling grid to identify a network of detectors, located at the centre of each sampling cell.

We associated each detector to 100 sub‐detectors, based on a 1 × 1 km grid constructed inside each cell, and projected each wolf detection to the closest sub‐detector. This allowed us to model wolf detection rates using a partially aggregated binomial observation process (Milleret et al., 2018), in which the number of wolf detections at a given detector was the result of a binomial process with 100 trials. From a temporal point of view, we aggregated all detections into a single sampling session, comprising the whole study period, thus modelling the temporal variation in capture probability through the spatial component of the detection process. In an SCR model, the detection process is controlled by two main parameters: *p*_*0*_ is the baseline capture probability, corresponding to the case in which a detector is located on the same location of an individual home range centre; *σ* is the distance from the home range centre at which capture probability is half of *p*_0_, and it is usually related to home range size (Efford, 2004). Using a half‐normal function to model capture probability attenuation, the resulting capture probability of individual *i* at detector *x* was:

$$p_{i,x}=p_{0,i,x}*\exp\left(-\frac{d_{i,x}^{2}}{2\sigma^{2}}\right)$$

where *d*_*i,x*_ was the linear distance between the activity centre of individual *i* and detector *x*.

To model variations in *p*_*0*_, we used a series of individual and spatial covariates, such as sampling effort (expressed as the total number of kilometres walked along transects in each cell), snow cover (defined as in the occupancy model), and the individual sex. We also added an individual random effect allowing a different intercept for each intensive area, to model local variations in capture probability. Finally, we used a finite‐mixture approach with two classes of heterogeneity to account for the residual differences in capture probability among wolves. To model variations in the *σ* parameter, we used the number of wild ungulate species available in each cell, similar to what was done for the occupancy model.

To account for the expected uneven distribution of wolves in the study area, we modelled the latent density as an inhomogeneous point process. For each location in the state space (the centre of each 10 × 10 cell), we expressed the expected abundance *λ* in cell *x* as a log‐linear function of a set of environmental covariates:

$$\;\log\left(\lambda_{x}\right)=\mu_{0}+\mu_{1}*{\mathrm{cov}}_{1}+\mu_{2}*{\mathrm{cov}}_{2}+\ldots \mu_{n}*{\mathrm{cov}}_{n}$$

where parameter μ_0_ is the density intercept and the remaining μ parameters are regression coefficients of the environmental covariates. Then, the expected population size was initially derived by integrating the *λ* values over the study area:

$$E\left(N\right)=\sum_{x=1}^{n}\lambda_{x}$$

We modelled variations in wolf density as a function of the habitat covariates used for the occupancy model, of the number of available wild ungulate prey, and added a spatial‐autocorrelation function which accounted for the spatial structure in the wolf population. Due to the wide latitudinal gradient in the study area, the effect of habitat covariates on wolf density was likely to be slightly different in the different portions of the sampling grid. Given the limitations imposed by sample size, we focused on the average large‐scale effects, but could not reveal local patterns in each of the 13 sampling areas.

To account for the undetected individuals, we augmented the observed dataset adding 10 times the number of detected individuals (Royle & Dorazio, 2012). Each individual *i* was considered being (*z*_*i*_ = 1) or not (*z*_*i*_ = 0) a member of the population according to a draw from a Bernoulli distribution of probability *ψ*, with *z*_*i*_ ~ *Bernoulli(ψ)*, where *ψ* was the probability for individual *i* to be a member of the population.

#### 2.4.3 Integration of the occupancy estimates into the SCR model

After building the SCR models, we integrated the information derived from the occupancy model into the SCR density function, with the objective of making the most out of the information that the presence/absence data could provide to density estimation. The main information we incorporated was that when a cell was estimated to be unoccupied by wolves, the local number of activity centres was set to zero. We obtained this constraint through the function “equals” in NIMBLE, which allowed to test the condition that the occupancy state of a given cell was unoccupied. Being *true_occ*_*i*_ the occupancy state of cell *i* (1 = occupied; 0 = unoccupied), we multiplied the density function in the SCR model by (1−equals(*true_occ*[*i*],0)). The function equals to zero when *true_occ*_*i*_ = 0, thus forcing density to be also zero, whereas it equals 1 when *true_occ*_*i*_ = 1, thus leaving the estimated density unmodified.

We ran all models with two Markov Chain Monte Carlo chains with 30,000 iterations each in the NIMBLE R package (de Valpine et al., 2015). We checked for convergence by calculating the *R‐hat* parameter (Gelman et al., 2013).

### 2.5 Simulations

After building and analysing the integrated spatial model, we also constructed two sets of Monte Carlo simulations, to assess the expected performance of our sampling and analytical design, and to guide a possible improvement of future applications. The main issue of our sampling design was that not all the area expected to be occupied by the wolf population was sampled (genetically or for presence signs). As a result, the population density estimates were only partially the result of capture‐recapture modelling in the sampled areas, as they were also produced through a density extrapolation from sampled to not sampled areas. The performance of such extrapolation needed to be evaluated, to assess if the proportion of study area sampled was sufficient to provide adequate statistical power and to produce unbiased estimates of total population abundance.

For the first set of simulations, we built a reduced study area, with a spatial extent of 29,000 km^2^ (19.8% of the actual sampling area) and divided it into 290 10 × 10 km cells. We generated the expected number of wolves in the simulated area using the results of the integrated spatial model and calculating the number corresponding to 19.8% of the estimated population size, thus simulating an average wolf density equal to the one estimated in the real model application. We distributed all the individual home range centres according to an inhomogeneous pattern, using a habitat suitability model produced by Boitani and Salvatori (2015) during the approval process of a new action plan for wolves in Italy. The model was based on four different techniques (GLM, GBM, ANN, MARS), whose predictions were averaged using the Ensemble Modelling method (Araújo & New, 2007). Individual wolves were divided by sex and assigned an individual detection probability, based on the results of the integrated spatial model. We then randomly identified 4 intensive sampling areas, made up of 9 cells and organized in a 3 × 3 spatial arrangement, which represented about the same proportion of sampled area as in the actual sampling design (12.4%). In addition, we randomly selected 30% of the cells for the presence signs sampling only, respecting the proportions adopted for the actual sampling.

After selecting all sampling cells, we simulated the sampling process using the same detection probability estimates obtained from the integrated spatial model. This generated a simulated dataset of presence signs and individual genetic detections. To test the performance of different modelling options, we analysed the simulated datasets using four different models: (i) the same integrated spatial model used for the actual abundance estimation; (ii) an SCR model with no data integration; (iii) an SCR model with no spatial autocorrelation function; (iv) an SCR model which did not account for sampling effort. We performed all the simulation in R 4.2.1 (R Development Core Tea, 2008) over 100 iterations and summarized the results calculating the relative bias and precision of the abundance estimates produced by each model in each iteration.

For the second set of simulations, we used the same design as the one illustrated above, but we varied the proportions of study area sampled both genetically and for presence signs. The aim of this second simulation was to compare the performance of different sampling designs and to identify the minimum proportion of study area to be sampled to obtain a pre‐defined level of bias and precision in the estimates. At each iteration, we selected the proportion of study area sampled for genetic sampling in the range 10%–50% and the proportion sampled for presence signs in the range 20%–50%. We ran 100 iterations using the integrated spatial model, and for each run, we estimated the associated bias and coefficient of variation of population size estimates. Then, we ran a first general linear model (GLM) with a Poisson distribution, using the rounded modulus of the relative bias of each iteration as response variable, whereas the proportions of study area sampled for genetic signs and presence signs were used as predictors. We used the same approach to run a second GLM with the same predictors and the coefficient of variation as response variable. The results of the two models allowed us to predict the minimum requirements of a future sampling design, in terms of what proportion of the study area should be sampled with both methods. We set the model performance goals to an absolute bias <10% and a CV < 10%.

## 3 RESULTS

### 3.1 Data collection

Out of the 2551 line transects initially selected, 2282 were covered at least once during the sampling period. The overall sampling effort along transects was 44,232 km, corresponding on average to 11,058 km travelled in each of the four sessions (±3824 SD; range 5338–13,233). Transect length was on average 4.75 km (SD = 2.77), whereas the average distance walked on transects in each cell was 20.57 km (SD = 15.19). We deployed a total of 598 photo traps. An average of 282.2 photo traps were active in each session (±101.3; range 147–379), corresponding to 9254.3 trap nights per session (±4591.8 SD; range 3128–13,388). In each cell, we deployed on average 2.64 photo traps (SD = 2.59), with an average of 63.03 (SD = 50.89) nights per trap.

Overall, we collected 15,993 wolf presence signs, including scats (*n* = 11,071), photos (*n* = 4285), tracks (*n* = 408), predation events (*n* = 97), dead wolves (*n* = 97), howlings (*n* = 11), hairs (*n* = 9), urine samples with blood (*n* = 8), saliva samples (*n* = 2), and other types of signs (*n* = 5; Table 2). Out of these, 1632 samples underwent genetic analysis for species and individual genotype identification (see below), including 1569 scats (1319 from intensive areas and 265 from extensive areas), 3 hair samples, 3 urine samples with blood, 30 muscle samples extracted from wolf carcasses, 3 blood samples, and a single saliva sample.

**TABLE 2:** Wolf presence data collected in south‐central Italy between October 2020 and April 2021 for each SCALP category.

Data type	C1	C2	C3	Total
Scats	886	10,185	—	11,071
Photos	4165	120	—	4285
Tracks	408	—	—	408
Predation events	—	2	95	97
Dead wolves	97	—	—	97
Howling	—	9	2	11
Hairs	—	—	9	9
Urine with blood	1	—	7	8
Saliva	2	—	—	2
Other	—	—	5	5
Total	5559	10,316	118	15,993

### 3.2 Genotype reconstruction and *taxon* identification

After the four to eight replicated PCR per sample per *locus* foreseen by the multiple‐tube protocol, 971 (61%) of the 1600 non‐invasively collected samples and all the 32 biological samples obtained from found dead or live‐trapped animals were successfully genotyped (*R* ≥ 0.990) at all biparental, uniparental and coding markers, corresponding to 622 individuals: 373 (180 females, 168 males, 25 with undetermined gender) wolves, 60 (26 females, 29 males, 5 with undetermined gender) recent wolf‐dog hybrids and 80 (25 females, 49 males, 5 with undetermined gender) introgressed wolves (for details see the Supporting Information).

### 3.3 Distribution and population size estimates

The probability to detect wolf presence during the sampling period was significantly affected by sampling effort. This was true both for wolf presence signs detected along transects (*β* = 2.71; 95% CIs = 2.49 to 2.93) and for the data derived from photo traps (*β* = 1.81; 95% CIs = 1.62 to 1.99). The effect of snow cover on wolf detection probability was only marginal (*β* = 0.09; 95% CIs = −0.03 to 0.22). The model also revealed that 43% of the sampling cells belonged to the group with the highest associated detection probability (P_2,H_ = 0.63; 95% CIs = 0.49 to 0.74), whereas the remaining 57% of the cells had a lower associated detection probability (P_2,L_ = 0.13; 95% CIs = 0.07 to 0.20). The probability to generate a false wolf detection by mistaking a dog scat for a wolf scat was on average 0.048 (95% CIs = 0.002 to 0.084), but the model was not able to detect significant geographical differences or to reveal the effect of any of the variables tested.

**FIGURE 2:**
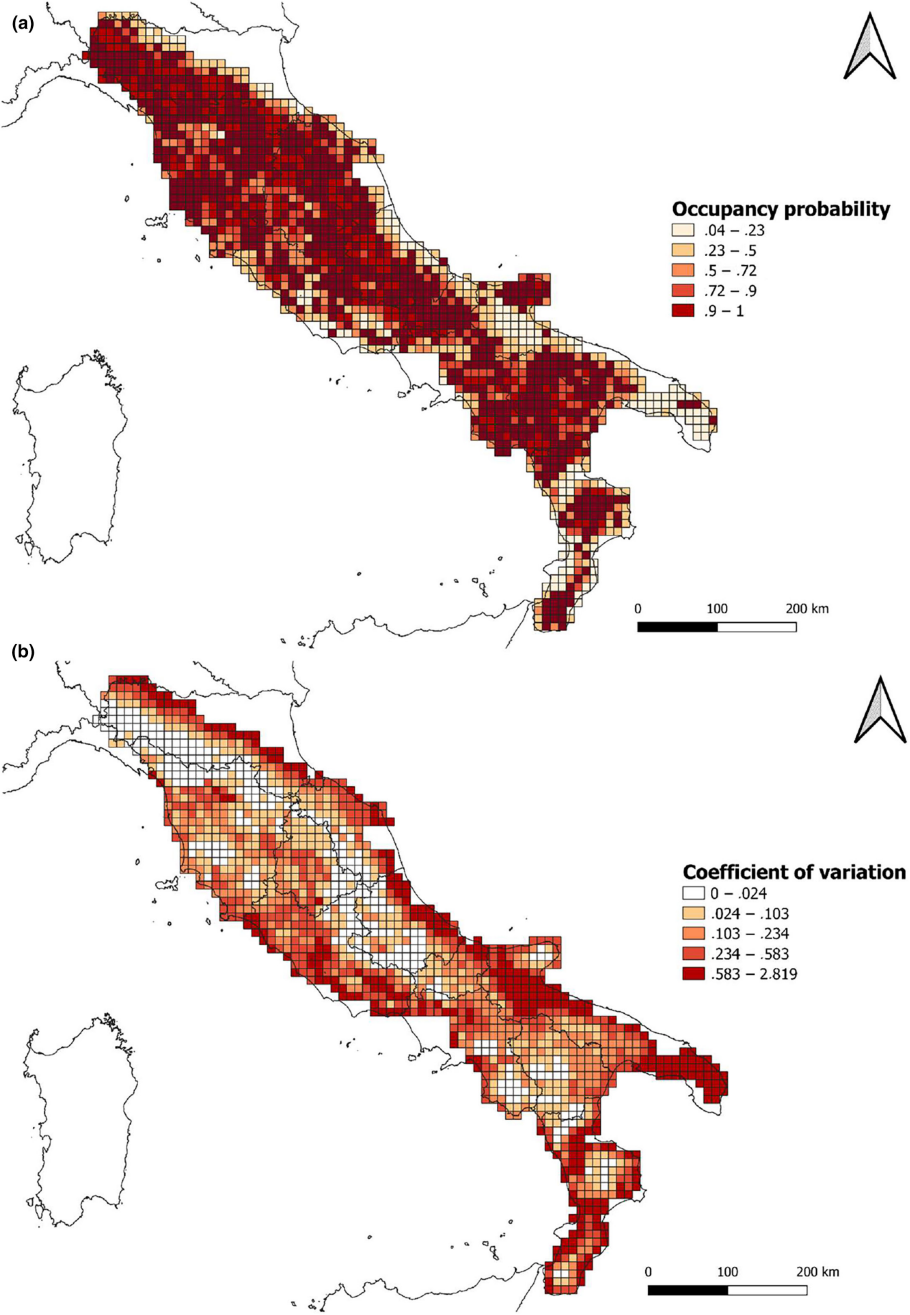
Cell‐specific wolf occupancy probability estimates (a) and the associated coefficient of variation (b) resulting from a sampling of presence signs and occupancy modelling in south‐central Italy, 2020–2021.

Wolf occupancy probability was significantly and positively affected by altitude (*β* = 1.14; 95% CIs = 0.51 to 1.79), forest cover (*β* = 0.71; 95% CIs = 0.06 to 1.40) and by the number of ungulate prey species (*β* = 0.93; 95% CIs = 0.37 to 1.53). The effects of human density (*β* = −040; 95% CIs = −0.93 to 0.05) and road density (*β* = −0.34; 95% CIs = −0.89 to 0.08) were both negative but only marginally significant. Overall, the model estimated that 74.2% of the study area (95% CIs = 70.5% to 77.9%) was occupied by the species, for a total extent of the wolf distribution of 108,534 km^2^ (95% CIs = 103,200 to 114,000). The cell‐specific occupancy probabilities are illustrated in Figure 2a, whereas the associated coefficients of variation of the occupancy estimates are shown in Figure 2b. The occupancy estimates were rather precise along the Apennines and in the core of the wolf distribution, whereas they were less precise at the periphery.

In the SCR part of the integrated spatial model, the individual baseline capture probability (*p*_*0*_) was positively correlated to sampling effort in each cell (*β* = 0.42; 95% CIs = 0.35 to 0.50), whereas sex and the amount of snow cover did not exhibit a significant effect on this parameter. Also, as expected in a species with a strong social structure as wolves, the data supported the existence of two groups of individuals in the population with different capture probabilities. Most of the individuals (83%; 95% CIs = 80% to 93%) were associated with the lower levels of capture probability (*p* = .0012, 95% CIs = 0.0003 to 0.0039), whereas the remaining 17% of the individuals (95% CIs = 7% to 20%) exhibited the highest capture probability estimates (*p* = .006, 95% CIs = 0.0025 to 0.0163), with a 5‐fold difference between the two groups. The number of wild ungulate prey species did not correlate significantly with the spatial parameter *σ*, whose average value was estimated at 3.49 km (95% CIs = 3.15 to 3.89). The estimated *σ* value was 2.8 times smaller than the average distance between detectors, close to the suggested range (1.5–2.5; Royle et al., 2014). This indicates that the trap spacing used for the SCR model is not expected to have introduced any relevant bias in population size estimates. Wolf local density was positively affected by altitude (*β* = 1.08; 95% CIs = 0.07 to 2.97) and by the extent of forest cover (*β* = 1.11; 95% CIs = 0.39 to 1.83), and negatively by human density (*β* = −0.76; 95% CIs = −2.39 to 0.94). The integrated spatial model produced an estimate of total population size for the Apennine wolf population of 2557 individuals (SD = 171.5; 95% CIs = 2127 to 2844), with an associated CV = 6.7%. When run alone, the SCR model produced a population size estimate of 2451 wolves (SD = 305.9; 95% CIs = 1939 to 3087), with an associated CV = 12.5%. By merging the two posterior distributions obtained in our study for the south‐central portion of the population and in Marucco et al. (2023) for the Alpine portion of the population, we estimated a total population size of 3501 wolves (SD = 249.5; 95% CIs = 2949 to 3945) in the whole country.

### 3.4 Simulations

**FIGURE 3:**
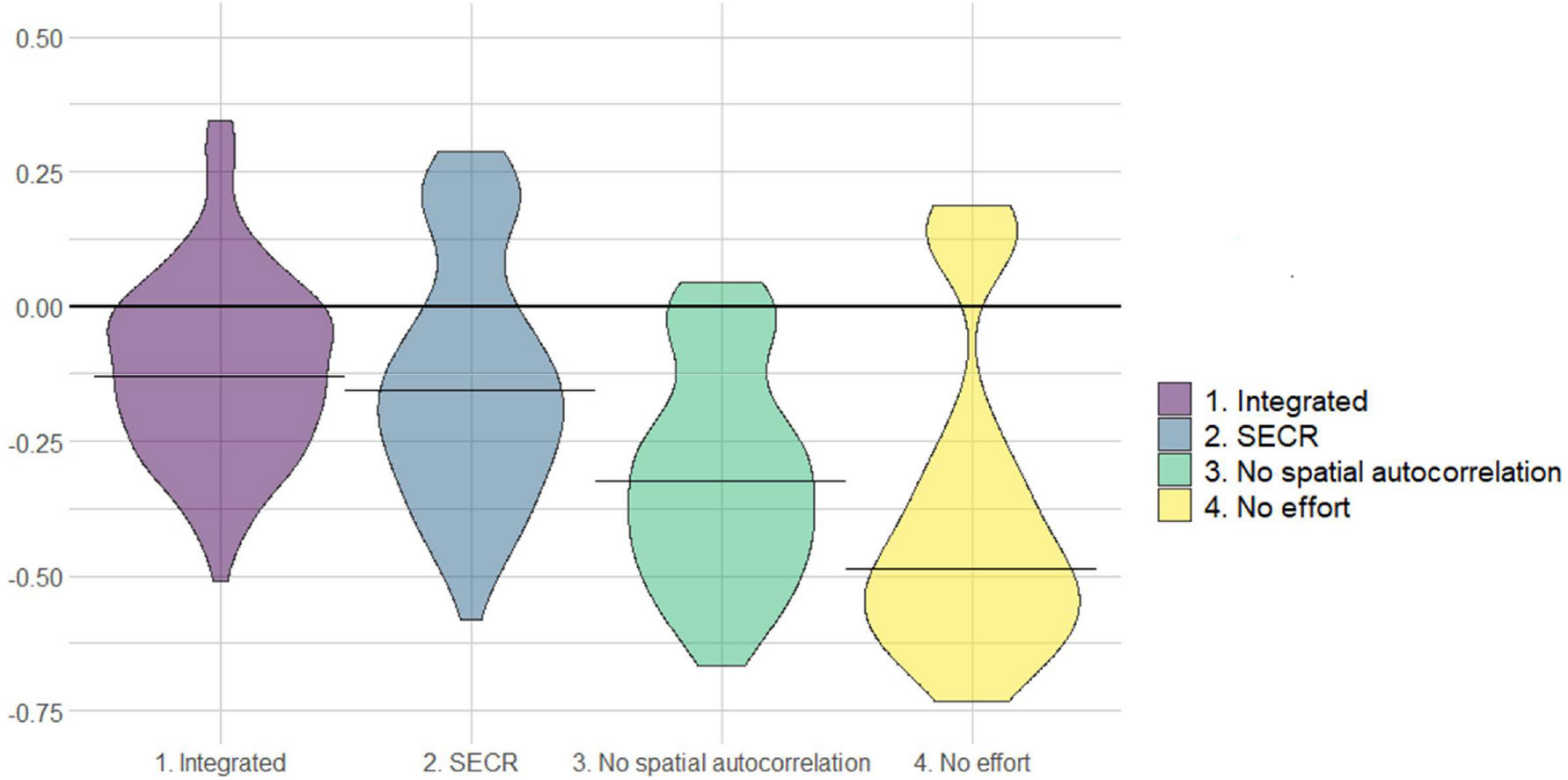
Relative bias in the estimation of population abundance, associated with four different capture‐recapture analytical designs. The data were derived from a set of simulated non‐invasive genetic sampling and presence signs sampling, resembling the field conditions of our wolf sampling project in south‐central Italy, 2020–2021.

In the first set of simulations, aimed at evaluating the performance of our design in terms of accuracy and precision of population size estimates, the integrated spatial model was associated with an average tendency to slightly underestimate population size (Figure 3). The average relative bias in the estimates was −0.129, with 64% of the estimates exhibiting a bias <20%. In terms of bias, the SCR model alone was also associated with a tendency to underestimate population size, with the average bias being −0.156 and 43% of the estimates exhibiting a bias <20% (Figure 3). The other two simulated scenarios performed poorly when compared to the first two. A model with no spatial autocorrelation function produced an average − 0.324 relative bias, whereas the model without any specification of sampling effort exhibited an average bias of −0.485 (Figure 3).

**FIGURE 4:**
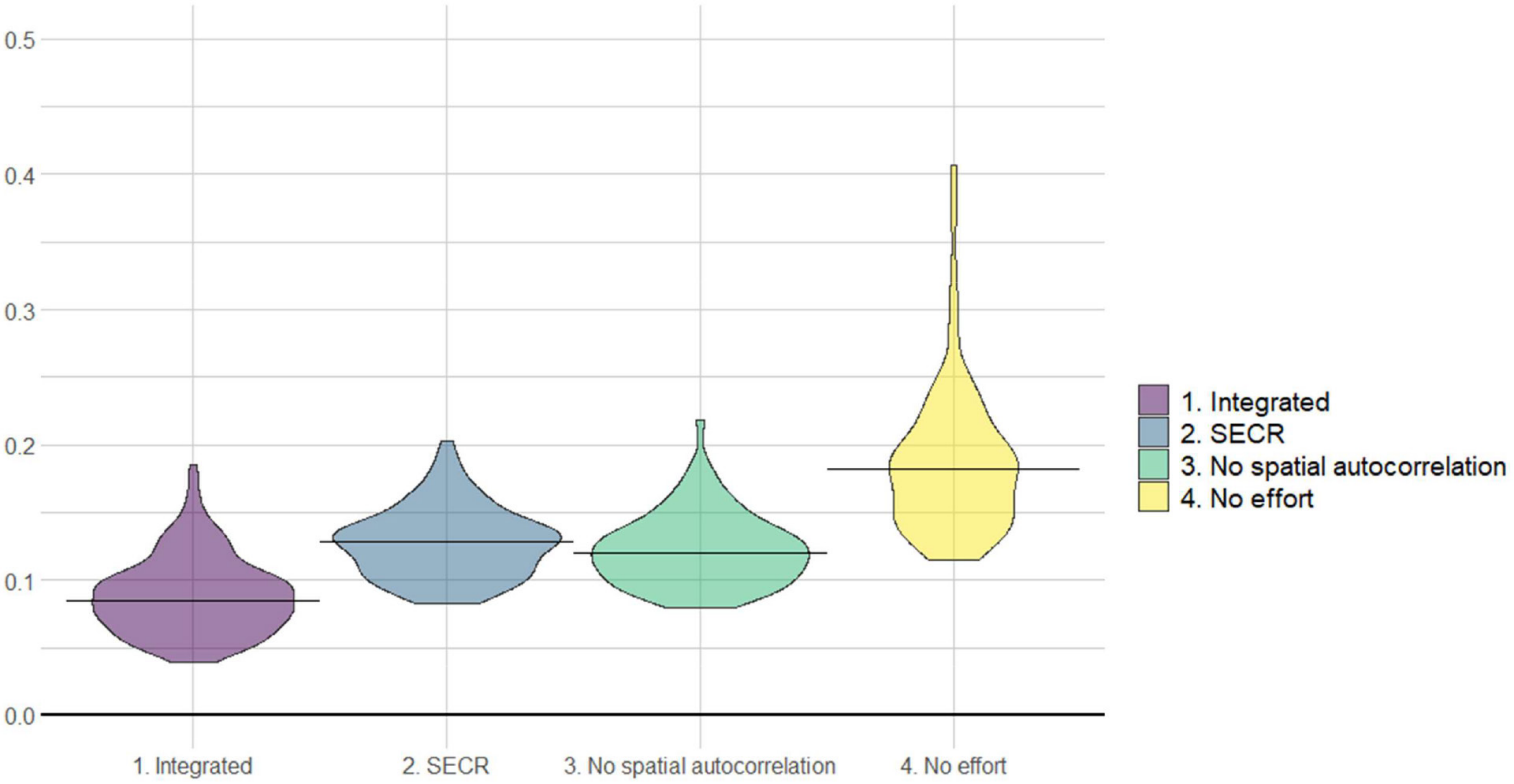
Precision in the estimation of population abundance, associated with four different capture‐recapture analytical designs. The data were derived from a set of simulated non‐invasive genetic sampling and presence signs sampling, resembling the field conditions of our wolf sampling project in south‐central Italy, 2020–2021.

In terms of precision of the estimates, the integrated spatial model exhibited the best performance among the four simulated designs. Its average associated coefficient of variation was 8.7%, with 70% of the estimates exhibiting a CV < 10% (Figure 4). The estimates produced by the SCR model alone were less precise, as the average coefficient of variation was 12.8% and only 12% of the estimates exhibited a CV < 10%. The model with no spatial autocorrelation exhibited similar performance than the SCR in terms of precision, whereas the model without effort data had an average CV = 18.2% (Figure 4).

**FIGURE 5:**
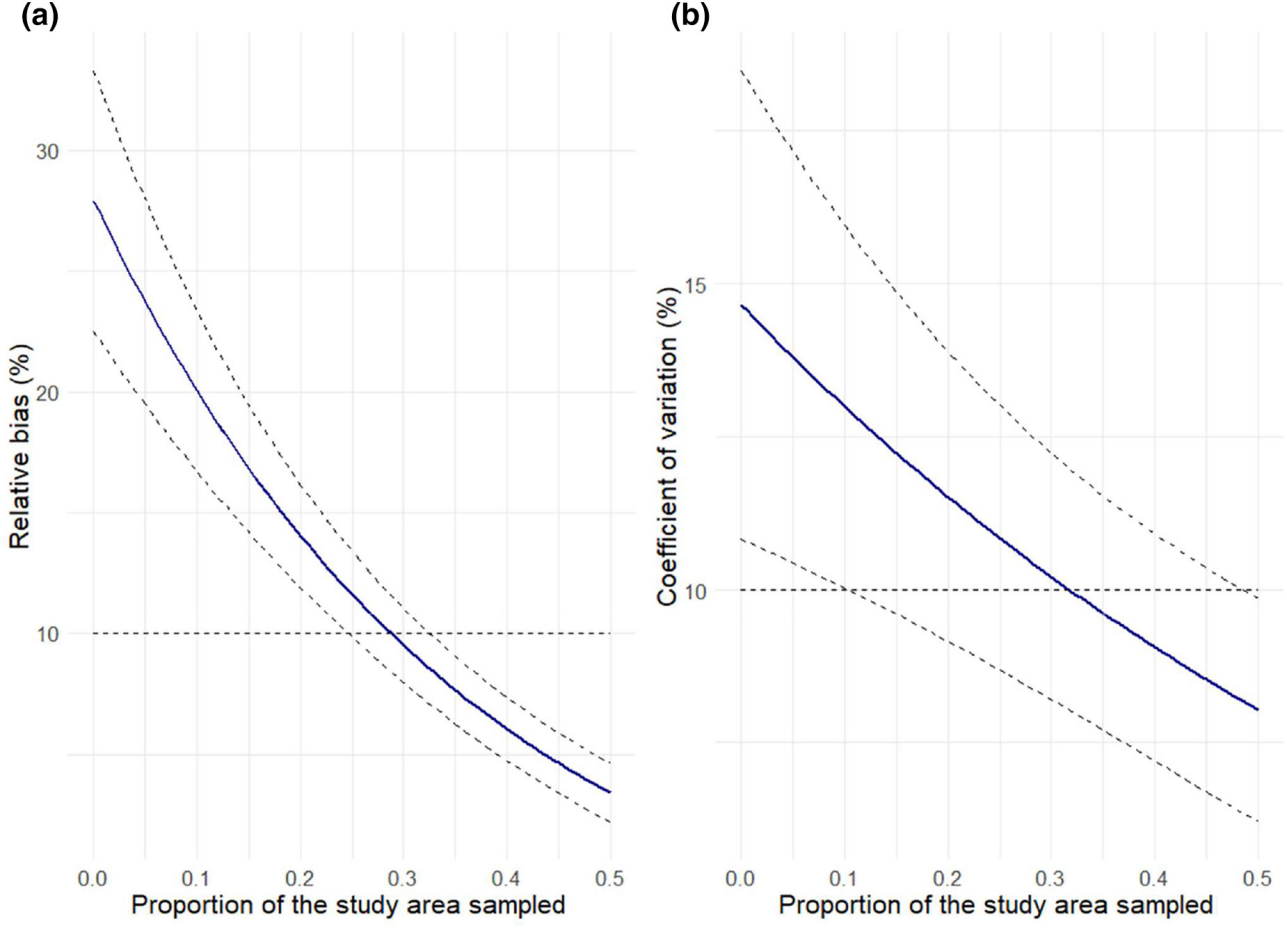
Expected accuracy (a) and precision (b) of population size estimates obtained when sampling 30% of the study area for presence signs and an increasing proportion for genetic samples.

Analysing the data derived from the second set of simulations, aimed at evaluating a possible improvement of sampling design for future applications, we found that the accuracy of population size estimates from an integrated design was significantly influenced by the percentage of study area sampled for genetic samples (*β* = −2.73; SE = 0.22), but not by the percentage sampled for presence signs (*β* = 0.09; SE = 0.29). As shown in Figure 5a, this generated the prediction that at least 30% of the study area should be selected for non‐invasive genetic samples to produce population size estimates with a satisfactory level of accuracy (bias < 10%). When running a GLM model on the simulated precision of the different sampling designs, we found that both the proportion selected for non‐invasive genetic samples (*β* = −1.20; SE = 0.29) and that selected for presence/absence sampling (*β* = −0.35; SE = 0.07) had a significant effect on the coefficient of variation associated with the estimates. The resulting predictions were in line with what was suggested by the analysis of model accuracy. When 30% of the area was sampled for presence signs, at least the same number of sampling cells should be genetically sampled, to produce population size estimates with a satisfactory level of precision (CV < 10%; Figure 5b).

## 4 DISCUSSION

### 4.1 Methodological implications

In term of performance, the main contribution of our integrated spatial modelling approach was to increase precision in the abundance estimates, whereas the combination of the SCR and occupancy models did not affect accuracy significantly (Figures 3 and 4). Other applications of integrated modelling for the estimation of demographic parameters produced similar conclusions. Tourani et al. (2020) developed and integrated spatial capture‐recapture (SCR) model, to incorporate multiple data sources with imperfect individual identification. They found that an integrated model outperformed a standard SCR model in terms of precision and, to a minor extent, accuracy, especially when detection probability was low and home ranges had a low degree of overlap (Tourani et al., 2020). In a study on the Louisiana black bear (*Ursus americanus luteolus*), Chandler and Clark (2014) also found that the main contribution of an integrated spatial model with respect to a standard SCR model was a reduction of variance in population size estimates. On one hand, this confirms that the inclusion of presence data, obtained through data sources which do not allow individual identification, can enhance the estimation of demographic parameters thanks to integrated spatial modelling. Species presence‐absence data have some advantages, with respect to individual recognition data. Being often collected through photo traps, visual observations, snow tracks, etc., and because they do not require genotyping, they usually generate larger datasets with a lower effort and costs than individual recognition techniques. Moreover, presence‐absence data often arise as by‐products of other field projects or are the result of incidental observations by citizens or hunters (Cretois et al., 2020; Ražen et al., 2020). On the other hand, our analysis and simulation exercise confirm that a robust and accurate estimation of demographic parameters still relies primarily on individual‐recognition data and capture‐recapture‐based techniques. Other analytical approaches, making use only of sampling techniques that do not allow individual identification, are being developed (Palencia et al., 2021; Rowcliffe et al., 2008), but simulation‐based assessments highlight that these methods are still very sensitive to external parameters, such as animal speed, and that their performance can change greatly with small variations in sampling conditions (Santini et al., 2022).

At this stage of research, therefore, SCR models are still the more powerful statistical tool available to produce robust and accurate density estimates, accounting for imperfect detection and spatial variation in sampling probabilities. While the main effort should still be to design and build a solid SCR model for population estimation, the possibility to integrate other data sources and increase precision is an important step forward, especially considering that unprecise estimates often have a poor value when informing management decisions. To this aim, our integration approach revealed both benefits and potential elements of improvement. Combining species presence data and the resulting occupancy model with a more structured dataset into an integrated occupancy‐SCR model offered the possibility to make the most out of the whole body of information resulting from our sampling effort (genetic data, presence signs, photos, etc.). The heterogeneity of the data types and sampling processes, though, introduced some elements of complexity during the integration phase. Occupancy and SCR models, in fact, are based on two very different underlying processes: one is based on grid‐based presence, the other on a point process referring to individuals, not to the species. This leaves some uncertainty in the degree of transferability of information between the two models, especially when using the estimated species absence to inform the SCR part of the model. Future improvements in this approach should go in the direction of using more similar underlying processes, such as in Tourani et al. (2020), thus also making the mathematical integration more straightforward. Still, simulations showed that the data integration did improve model performance and that the potential bias induced by data integration was probably minimal (Figure 3). Underestimation is a known risk, especially when estimating density for gregarious species. Bischof, Dupont, et al. (2020) and Bischof, Milleret, et al. (2020) used simulation tools to show that large group size and high level of cohesiveness can be a source of bias and reduce precision in SCR‐based population size estimates; Jiménez et al. (2023) also revealed that gregariousness in wolves can induce overdispersion and cause bias, if not properly accounted for.

When projecting our first wolf estimation project into a future national population monitoring plan, the simulation work showed that the area subject to NGS should be increased to further improve the accuracy of population size estimates and avoid the risk of underestimation. At least 30% of the wolf distribution should be intensively sampled for individual‐recognition data, while at least a similar proportion should be sampled for presence‐absence data. This suggests that, while it is feasible to produce reliable population size estimates without having to sample 100% of a species distribution, a careful a priori evaluation should be done to identify the optimal sampling design. SCR models, in this sense, are a powerful tool. They can allocate activity centres of sampled individuals not only within the sampled area but also outside of it, thus generating density estimates also in the portions of a species distribution buffering the actual sampling grid. On the other hand, such extrapolation becomes gradually less reliable when moving away from sampled areas. Identifying the correct proportion of the species distribution to be sampled and the spatial arrangement of all the sampled areas is, therefore, crucial to avoid that density estimation might suffer from a lack of information in some portions of the study area. Survey effort is always constrained by available resources, but spatially balanced sampling and stratification can help to appropriately distribute it and are key approaches for improving the representativeness of the sample and the precision of estimates (Perret et al., 2022; Stevens & Olsen, 2004; Thompson, 2012). In our case, simulations showed that sampling 30% of the area or more would be a good minimum compromise between accuracy, precision, and sampling effort. This approach could be extended and applied to other species and geographic regions, but specific a priori evaluations of model requirements and expected performance should be made.

### 4.2 Management implications

The estimates resulting from our work represent the first formal assessment of the wolf distribution and abundance in the regions of south‐central Italy. Combined with the ones produced for the alpine regions (Marucco et al., 2023), this also provides the first estimate for the entire country, which will represent a fundamental baseline for future assessments of population trend, and to inform management actions. Our work confirmed that wolves in south‐central Italy are on their way to occupy most of their suitable habitat, well outside the habitat types of broad‐leaved mountain forest, traditionally considered as the election environment for the species (Mech & Boitani, 2019). As shown in Figure 2a, while the Apennine Mountain chain remains the backbone of wolf distribution, high occupancy probabilities (and confirmed presence signs) exist also in coastal areas and in the plains associated with higher levels of human density and infrastructures. This represents a further step in a successful conservation story, if we consider that, only a few decades ago, wolves in Italy were on the verge of extinction (Zimen & Boitani, 1975). It also raises the issue of the complex land sharing with human activities and the impacts it may cause. While the more traditional forms of wolf impact, such as depredation on livestock, remain an issue and a source of social conflicts (Gervasi et al., 2021, 2022), wolves in newly colonized and densely inhabited areas are more likely to generate different forms of negative interactions (Carter & Linnell, 2016), such as predation on domestic dogs and other pets (Iliopoulos et al., 2021; Kojola et al., 2023), aggressive interactions with humans (Linnell et al., 2021), etc. Accordingly, the reports of wolf‐killed dogs and of not fearful behaviours by wolves towards humans have been increasing in recent years (ISPRA, unpublished data). In such a context, the traditional approach, centred on national parks and marginal mountain areas as the main actors and targets of management and conservation actions is no more in line with the ecological reality of the wolf population. Our study provides a methodologically sound picture of this situation (Figure 2a), which is in line with the expansion patterns of wolf populations in several other European countries (Eriksson & Dalerum, 2018; Louvrier et al., 2018). An effective monitoring of these new forms of human‐wolf interactions should be enhanced, to better understand their dynamics, possible causes and predictors, and to design effective management actions. Also, the establishment of wolves in new habitat types, closer to human settlements, will require a gradual behavioural co‐adaptation (Carter & Linnell, 2016). Humans will need to increase their awareness about wolf presence and to modify some of their behaviours, accordingly. Management actions should be put in place to reduce the risks of human‐wolf encounters, for instance by reducing food availability in urban and periurban areas, and to promote active avoidance of humans by wolves.

On the other hand, wolves in newly colonized and densely inhabited areas are more likely to be subject to human‐related mortality risks, such as road accidents, poaching, poisoning, etc. These risks have been already highlighted as the main sources of wolf mortality in Italy, and therefore likely to be strong drivers of wolf population dynamics also in future years (Musto et al., 2021). The value of a first national assessment, as the one resulting from this work and from Marucco et al. (2023), is to place a first stepstone towards the implementation of a national monitoring program, based on periodic surveys of the wolf population and with the aim of detecting not only population trends but also to explore the patterns and causes of wolf mortality, reproduction and wolf‐dog hybridization rates and the other fundamental parameters needed to build a reliable population model. The need for such a tool is even more crucial during this transition period, in which a shift from a purely protective to a more active approach (including lethal control) is occurring in wolf management and conservation. Italy shares this shift and its challenges with several other European countries. To this aim, our simulation work provides a quantitative basis to help managers and researchers find a good balance between the requirements of the statistical tools and the limitations of the available resources, when setting up a long‐term monitoring program for wide‐ranging elusive species (see below for more details).

The criteria to classify the unknown detected genotypes at the *taxon* level (Caniglia et al., 2020) allowed us to also investigate the presence of anthropogenic wolf‐dog hybridization in the intensively sampled areas. About 10% of the wild individuals sampled showed recent wolf‐dog admixture signals within the first two to three hybridization generations, whereas about 13% of them showed introgression signs older than three backcrossing generations in the past. These proportions compare well with the results extrapolated from the analyses of a large sample of putative free‐living wolves collected in Italy during the last 20 years (Caniglia et al., 2020). However, most of the data about wolf‐dog hybridization were obtained by genotyping the biological samples at a reduced number of molecular markers, which, although highly differentiating between dogs and wolves (Caniglia et al., 2013), represent only a moderately resolved snapshot of the non‐coding variability observable within the whole canine genome (Galaverni et al., 2016). Therefore, they can only provide preliminary evidence of the phenomenon which surely merits further detailed multidisciplinary investigations to ensure reliable prevalence estimates at the national scale (Caniglia et al., 2020).

## AUTHOR CONTRIBUTIONS

**Vincenzo Gervasi:** Formal analysis (lead); methodology (equal); writing – original draft (lead); writing – review and editing (lead). **Paola Aragno:** Data curation (equal); methodology (equal); project administration (equal); supervision (equal); writing – review and editing (equal). **Valeria Salvatori:** Conceptualization (equal); methodology (equal); project administration (equal); writing – review and editing (equal). **Romolo Caniglia:** Formal analysis (equal); writing – review and editing (equal). **Daniele De Angelis:** Data curation (equal); formal analysis (supporting); writing – review and editing (equal). **Elena Fabbri:** Formal analysis (equal); writing – review and editing (equal). **Valentina La Morgia:** Conceptualization (equal); methodology (equal); supervision (equal); writing – original draft (equal); writing – review and editing (equal). **Francesca Marucco:** Conceptualization (equal); methodology (equal); writing – review and editing (equal). **Edoardo Velli:** Formal analysis (equal); writing – review and editing (equal). **Piero Genovesi:** Conceptualization (equal); funding acquisition (equal); supervision (equal); writing – review and editing (equal).

## FUNDING INFORMATION

This research was funded by The Italian Ministry of the Environment and Energetic Security, as part of an agreement with ISPRA for the “National monitoring activities included in the Wolf Action Plan.”

## CONFLICT OF INTEREST STATEMENT

The authors certify that they have no affiliations with or involvement in any organization or entity with any financial interest or non‐financial interest in the subject matter or materials discussed in this manuscript.

## Supporting information


Data S1.

## Data Availability

Data are available from the SINANET repository: https://sinacloud.isprambiente.it/portal/apps/webappviewer/index.html?id=649149e973894b788e9053baeb9bdc99.

## References

[ece311285-bib-0001] Allan, B. M. , Nimmo, D. G. , Ierodiaconou, D. , VanDerWal, J. , Koh, L. P. , & Ritchie, E. G. (2018). Futurecasting ecological research: The rise of technoecology. Ecosphere, 9, e02163.

[ece311285-bib-0002] Araújo, M. B. , & New, M. (2007). Ensemble forecasting of species distributions. Trends in Ecology & Evolution, 22, 42–47. 17011070 10.1016/j.tree.2006.09.010

[ece311285-bib-0003] Beng, K. C. , & Corlett, R. T. (2020). Applications of environmental DNA (eDNA) in ecology and conservation: Opportunities, challenges and prospects. Biodiversity and Conservation, 29, 2089–2121.

[ece311285-bib-0004] Besnier, F. , & Glover, K. A. (2013). Parallel structure: A R package to distribute parallel runs of the population genetics program structure on multi‐core computers. PLoS One, 8, e70651. 23923012 10.1371/journal.pone.0070651 PMC3726640

[ece311285-bib-0005] Bischof, R. , Dupont, P. , Milleret, C. , Chipperfield, J. , & Royle, J. A. (2020). Consequences of ignoring group association in spatial capture–recapture analysis. Wildlife Biology, 2020, 1–10.

[ece311285-bib-0006] Bischof, R. , Milleret, C. , Dupont, P. , Chipperfield, J. , Tourani, M. , Ordiz, A. , de Valpine, P. , Turek, D. , Royle, J. A. , Gimenez, O. , Flagstad, Ø. , Åkesson, M. , Svensson, L. , Brøseth, H. , & Kindberg, J. (2020). Estimating and forecasting spatial population dynamics of apex predators using transnational genetic monitoring. Proceedings of the National Academy of Sciences of the United States of America, 117, 30531–30538. 33199605 10.1073/pnas.2011383117 PMC7720137

[ece311285-bib-0007] Blanc, L. , Marboutin, E. , Gatti, S. , Zimmermann, F. , & Gimenez, O. (2014). Improving abundance estimation by combining capture‐recapture and occupancy data: Example with a large carnivore. Journal of Applied Ecology, 51, 1733–1739.

[ece311285-bib-0008] Boitani, L. , & Salvatori, V. (2015). Piano di conservazione e gestione del lupo in Italia. Report for the Italian Ministry of the Environment. pp. 1–59. (In Italian).

[ece311285-bib-0010] Caniglia, R. , Fabbri, E. , Greco, C. , Galaverni, M. , Manghi, L. , Boitani, L. , Sforzi, A. , & Randi, E. (2013). Black coats in an admixed wolf × dog pack is melanism an indicator of hybridization in wolves? European Journal of Wildlife Research, 59, 543–555.

[ece311285-bib-0011] Caniglia, R. , Fabbri, E. , Galaverni, M. , Milanesi, P. , & Randi, E. (2014). Non‐invasive sampling and genetic variability, pack structure, and dynamics in an expanding wolf population. Journal of Mammalogy, 95, 41–59.

[ece311285-bib-0012] Caniglia, R. , Galaverni, M. , Delogu, M. , Fabbri, E. , Musto, C. , & Randi, E. (2016). Big bad wolf or man's best friend? Unmasking a false wolf aggression on humans. Forensic Science International: Genetics, 24, e4–e6. 27353864 10.1016/j.fsigen.2016.06.009

[ece311285-bib-0013] Caniglia, R. , Galaverni, M. , Velli, E. , Mattucci, F. , Canu, A. , Apollonio, M. , Mucci, N. , Scandura, M. , & Fabbri, E. (2020). A standardized approach to empirically define reliable assignment thresholds and appropriate management categories in deeply introgressed populations. Scientific Reports, 10, 2862. 32071323 10.1038/s41598-020-59521-2 PMC7028925

[ece311285-bib-0014] Carter, N. H. , & Linnell, J. D. C. (2016). Co‐adaptation is key to coexisting with large carnivores. Trends in Ecology & Evolution, 31, 575–578. 27377600 10.1016/j.tree.2016.05.006

[ece311285-bib-0015] Chandler, R. B. , & Clark, J. D. (2014). Spatially explicit integrated population models. Methods in Ecology and Evolution, 5, 1351–1360.

[ece311285-bib-0016] Chapron, G. , Kaczensky, P. , Linnell, J. D. C. , von Arx, M. , Huber, D. , Andrén, H. , López‐Bao, J. V. , Adamec, M. , Álvares, F. , Anders, O. , Balčiauskas, L. , Balys, V. , Bedő, P. , Bego, F. , Blanco, J. C. , Breitenmoser, U. , Brøseth, H. , Bufka, L. , Bunikyte, R. , … Boitani, L. (2014). Recovery of large carnivores in Europe's modern human‐dominated landscapes. Science, 346, 1517–1519. 25525247 10.1126/science.1257553

[ece311285-bib-0017] Cretois, B. , Linnell, J. D. C. , Grainger, M. , Nilsen, E. B. , & Rød, J. K. (2020). Hunters as citizen scientists: Contributions to biodiversity monitoring in Europe. Global Ecology and Conservation, 23, e01077.

[ece311285-bib-0018] de Valpine, P. , Turek, D. , Paciorek, C. J. , Anderson‐Bergman, C. , Lang, D. T. , & Bodik, R. (2015). Programming with models: Writing statistical algorithms for general model structures with NIMBLE. Journal of Computational and Graphical Statistics, 26, 403–413.

[ece311285-bib-0019] Dupont, P. , Milleret, C. , Gimenez, O. , & Bischof, R. (2019). Population closure and the bias‐precision trade‐off in spatial capture–recapture. Methods in Ecology and Evolution, 10, 661–672.

[ece311285-bib-0020] Efford, M. (2004). Density estimation in live‐trapping studies. Oikos, 106, 598–610.

[ece311285-bib-0021] Eriksson, T. , & Dalerum, F. (2018). Identifying potential areas for an expanding wolf population in Sweden. Biological Conservation, 220, 170–181.

[ece311285-bib-0022] Fabbri, E. , Miquel, C. , Lucchini, V. , Santini, A. , Caniglia, R. , Duchamp, C. , Weber, J. M. , Lequette, B. , Marucco, F. , Boitani, L. , Fumagalli, L. , Taberlet, P. , & Randi, E. (2007). From the Apennines to the Alps: Colonization genetics of the naturally expanding Italian wolf (*Canis lupus*) population. Molecular Ecology, 16, 1661–1671. 17402981 10.1111/j.1365-294X.2007.03262.x

[ece311285-bib-0023] Fabbri, E. , Velli, E. , D'Amico, F. , Galaverni, M. , Mastrogiuseppe, L. , Mattucci, F. , & Caniglia, R. (2018). From predation to management: Monitoring wolf distribution and understanding depredation patterns from attacks on livestock. Hystrix, Italian Journal of Mammalogy, 29, 101–110.

[ece311285-bib-0024] Galaverni, M. , Caniglia, R. , Fabbri, E. , Milanesi, P. , & Randi, E. (2016). One, no one, or one hundred thousand: How many wolves are there currently in Italy? Mammal Research, 61, 13–24.

[ece311285-bib-0025] Gelman, A. , Carlin, J. B. , Stern, H. S. , Dunson, D. B. , Vehtari, A. , & Rubin, D. B. (2013). Bayesian data analysis. Chapman and Hall/CRC.

[ece311285-bib-0026] Gervasi, V. , Salvatori, V. , Catullo, G. , & Ciucci, P. (2021). Assessing trends in wolf impact on livestock through verified claims in historical vs. recent areas of occurrence in Italy. Europena Journal of Wildlife Research, 67, 82.

[ece311285-bib-0027] Gervasi, V. , Zingaro, M. , Aragno, P. , Genovesi, P. , & Salvatori, V. (2022). Stima dell'impatto del lupo sulle attività zootecniche in Italia. Analisi del periodo 2015–2019. ISPRA. Report for the Italian Ministry of the Environment. (In Italian).

[ece311285-bib-0028] Iliopoulos, Y. , Antoniadi, E. , Kret, E. , Zakkak, S. , & Skartsi, T. (2021). Wolf–hunting dog interactions in a biodiversity hot spot area in northern Greece: Preliminary assessment and implications for conservation in the Dadia‐Lefkimi‐Soufli Forest National Park and adjacent areas. Animals, 11, 3235. 34827967 10.3390/ani11113235 PMC8614248

[ece311285-bib-0029] Jiménez, J. , Cara, D. , García‐Dominguez, F. , & Barasona, J. A. (2023). Estimating wolf (*Canis lupus*) densities using video camera traps and spatial capture–recapture analysis. Ecosphere, 14, e4604.

[ece311285-bib-0030] Kendall, W. L. , Hines, J. E. , Nichols, J. D. , & Grant, E. H. C. (2013). Relaxing the closure assumption in occupancy models: Staggered arrival and departure times. Ecology, 94, 610–617. 23687887 10.1890/12-1720.1

[ece311285-bib-0031] Kéry, M. , & Royle, J. A. (2016). Applied hierarchical modeling in ecology: Analysis of distribution, abundance and species richness in R and BUGS. Volume 2: Dynamic and advanced models. Academic Press.

[ece311285-bib-0032] Kojola, I. , Hallikainen, V. , Nivala, V. , Heikkinen, S. , Tikkunen, M. , Huhta, E. , Ruha, L. , & Pusenius, J. (2023). Wolf attacks on hunting dogs are negatively related to prey abundance in Finland: An analysis at the wolf territory level. European Journal of Wildlide Research, 69, 26.

[ece311285-bib-0033] Lauret, V. , Labach, H. , Authier, M. , & Gimenez, O. (2021). Using single visits into integrated occupancy models to make the most of existing monitoring programs. Ecology, 102, e03535. 34514594 10.1002/ecy.3535

[ece311285-bib-0034] Lauret, V. , Labach, H. , Turek, D. , Laran, S. , & Gimenez, O. (2023). Integrated spatial models foster complementarity between monitoring programmes in producing large‐scale bottlenose dolphin indicators. Animal Conservation, 26, 228–238.

[ece311285-bib-0035] Linnell, J. D. C. , Cretois, B. , Nilsen, E. B. , Rolandsen, C. M. , Solberg, E. J. , Veiberg, V. , Kaczensky, P. , van Moorter, B. , Panzacchi, M. , Rauset, G. R. , & Kaltenborn, B. (2020). The challenges and opportunities of coexisting with wild ungulates in the human‐dominated landscapes of Europe's Anthropocene. Biological Conservation, 244, 108500.

[ece311285-bib-0036] Linnell, J. D. C. , Kovtun, E. , & Rouart, I. (2021). Wolf attacks on humans: An update for 2002–2020. NINA Report 1944 Norwegian Institute for Nature Research.

[ece311285-bib-0037] López‐Bao, J. V. , Godinho, R. , Pacheco, C. , Lema, F. J. , García, E. , Llaneza, L. , Palacios, V. , & Jiménez, J. (2018). Toward reliable population estimates of wolves by combining spatial capture‐recapture models and non‐invasive DNA monitoring. Scientific Reports, 8, 2177. 29391588 10.1038/s41598-018-20675-9 PMC5794931

[ece311285-bib-0038] Louvrier, J. , Duchamp, C. , Lauret, V. , Marboutin, E. , Cubaynes, S. , Choquet, R. , Miquel, C. , & Gimenez, O. (2018). Mapping and explaining wolf recolonization in France using dynamic occupancy models and opportunistic data. Ecography, 41, 647–660.

[ece311285-bib-0039] Mackenzie, D. I. , Nichols, J. D. , Lachman, G. B. , Droege, S. , Royle, J. A. , & Langtimm, C. A. (2002). Estimating site occupancy rates when detection probabilities are less than one. Ecology, 83, 2248–2255.

[ece311285-bib-0040] Marucco, F. , Boiani, M. V. , Dupont, P. , Milleret, C. , Avanzinelli, E. , Pilgrim, K. , Schwartz, M. K. , von Hardenberg, A. , Perrone, D. S. , Friard, O. P. , Menzano, A. , Bisi, F. , Fattori, U. , Tomasella, M. , Calderola, S. , Carolfi, S. , Ferrari, P. , Chioso, C. , Truc, F. , … Bischof, R. (2023). A multidisciplinary approach to estimating wolf population size for long‐term conservation. Conservation Biology, 37, e14132. 37259636 10.1111/cobi.14132

[ece311285-bib-0041] McDonald, L. L. (2004). Sampling rare populations. In W. L. Thompson (Ed.), Sampling rare and elusive species (pp. 11–42). Island Press.

[ece311285-bib-0042] Mech, D. , & Boitani, L. (2019). Wolves: Behavior, ecology, and conservation. University of Chicago Press.

[ece311285-bib-0043] Miller, C. R. , Joyce, P. , & Waits, L. P. (2002). Assessing allelic dropout and genotype reliability using maximum likelihood. Genetics, 160, 357–366. 11805071 10.1093/genetics/160.1.357 PMC1461941

[ece311285-bib-0044] Miller, D. A. , Nichols, J. D. , Mcclintock, B. T. , Grant, E. H. , Bailey, L. L. , & Weir, L. A. (2011). Improving occupancy estimation when two types of observational error occur: Non‐detection and species misidentification. Ecology, 92, 1422–1428. 21870616 10.1890/10-1396.1

[ece311285-bib-0045] Milleret, C. , Dupont, P. , Brøseth, H. , Kindberg, J. , Royle, J. A. , & Bischof, R. (2018). Using partial aggregation in spatial capture recapture. Methods in Ecology and Evolution, 9, 1896–1907.

[ece311285-bib-0046] Molinari‐Jobin, A. , Wölfl, S. , Marboutin, E. , Molinari, P. , Woelfl, M. , Kos, I. , Fasel, M. , Koren, I. , Fuxjäger, C. , Breitenmoser, C. , Huber, T. , Blazic, M. , & Breitenmoser, U. (2012). Monitoring the lynx in the Alps. Hystrix, 23, 49–53.

[ece311285-bib-0047] Musto, C. , Cerri, J. , Galaverni, M. , Caniglia, R. , Fabbri, E. , Mucci, N. , Apollonio, M. , Bonilauri, P. , Maioli, G. , & Fontana, M. C. (2021). Men and wolves: Anthropogenic causes are an important driver of wolf mortality in human‐dominated landscapes in Italy. Global Ecology and Conservation, 32, e01892.

[ece311285-bib-0048] Oliver, R. Y. , Iannarilli, F. , Ahumada, J. , Fegraus, E. , Flores, N. , Kays, R. , Birch, T. , Ranipeta, A. , Rogan, M. S. , Sica, Y. V. , & Jetz, W. (2023). Camera trapping expands the view into global biodiversity and its change. Philosophical Transactions of the Royal Society, B: Biological Sciences, 378, 20220232. 10.1098/rstb.2022.0232 PMC10225860 37246379

[ece311285-bib-0049] Pacifici, K. , Reich, B. J. , Miller, D. A. W. , Gardner, B. , Stauffer, G. , Singh, S. , McKerrow, A. , & Collazo, J. A. (2017). Integrating multiple data sources in species distribution modeling: A framework for data fusion. Ecology, 98, 840–850. 28027588 10.1002/ecy.1710

[ece311285-bib-0050] Palencia, P. , Rowcliffe, J. M. , Vicente, J. , & Acevedo, P. (2021). Assessing the camera trap methodologies used to estimate density of unmarked populations. Journal of Applied Ecology, 58, 1583–1592.

[ece311285-bib-0051] Peakall, R. , & Smouse, P. E. (2012). GenAlEx 6.5: Genetic analysis in excel. Population genetic software for teaching and research—An update. Bioinformatics, 28, 2537–2539. 22820204 10.1093/bioinformatics/bts460 PMC3463245

[ece311285-bib-0052] Perret, J. , Charpentier, A. , Pradel, R. , Papuga, G. , & Besnard, A. (2022). Spatially balanced sampling methods are always more precise than random ones for estimating the size of aggregated populations. Methods in Ecology and Evolution, 13, 2743–2756.

[ece311285-bib-0053] Pledger, S. (2005). The performance of mixture models in heterogeneous closed population capture–recapture. Biometrics, 61, 868–873. 16135042 10.1111/j.1541-020X.2005.00411_1.x

[ece311285-bib-0054] Popescu, V. D. , Iosif, R. , Pop, M. I. , Chiriac, S. , Bouroș, G. , & Furnas, B. J. (2017). Integrating sign surveys and telemetry data for estimating brown bear (*Ursus arctos*) density in the Romanian Carpathians. Ecology and Evolution, 7, 7134–7144. 28944005 10.1002/ece3.3177 PMC5606905

[ece311285-bib-0055] R Development Core Tea . (2008). R: A language and environment for statistical computing. R Foundation for Statistical Computing.

[ece311285-bib-0056] Ražen, N. , Kuralt, Ž. , Fležar, U. , Bartol, M. , Černe, R. , Kos, I. , Krofel, M. , Luštrik, R. , Skrbinšek, A. M. , & Potočnik, H. (2020). Citizen science contribution to national wolf population monitoring: What have we learned? European Journal of Wildlife Research, 66, 46.

[ece311285-bib-0057] Rowcliffe, J. M. , Field, J. , Turvey, S. T. , & Carbone, C. (2008). Estimating animal density using camera traps without the need for individual recognition. Journal of Applied Ecology, 45, 1228–1236.

[ece311285-bib-0058] Royle, J. A. , Chandler, R. B. , Sollmann, R. , & Gardner, B. (2014). Spatial capture‐recapture. Elsevier.

[ece311285-bib-0059] Royle, J. A. , & Dorazio, R. M. (2012). Parameter‐expanded data augmentation for Bayesian analysis of capture–recapture models. Journal of Ornithology, 152, 521–537.

[ece311285-bib-0060] Santini, G. , Abolaffio, M. , Ossi, F. , Franzetti, B. , Cagnacci, F. , & Focardi, S. (2022). Population assessment without individual identification using camera‐traps: A comparison of four methods. Basic and Applied Ecology, 61, 68–81.

[ece311285-bib-0062] Schad, L. , & Fischer, J. (2022). Opportunities and risks in the use of drones for studying animal behaviour. Methods in Ecology and Evolution, 14, 1864–1882.

[ece311285-bib-0063] Schaub, M. , & Abadi, F. (2011). Integrated population models: A novel analysis framework for deeper insights into population dynamics. Journal of Ornithology, 152, S227–S237.

[ece311285-bib-0064] Stevens, D. L. , & Olsen, A. R. (2004). Spatially balanced sampling of natural resources. Journal of the American Statistical Association, 99, 262–278.

[ece311285-bib-0065] Thompson, S. K. (2012). Sampling. Third Edition. Wiley series in probability and statistics. John Wiley & Sons Inc.

[ece311285-bib-0066] Tourani, M. (2022). A review of spatial capture–recapture: Ecological insights, limitations, and prospects. Ecology and Evolution, 12, e8468. 35127014 10.1002/ece3.8468 PMC8794757

[ece311285-bib-0067] Tourani, M. , Dupont, P. , Nawaz, M. A. , & Bischof, R. (2020). Multiple observation processes in spatial capture–recapture models: How much do we gain? Ecology, 101, e03030. 32112415 10.1002/ecy.3030

[ece311285-bib-0068] Valière, N. (2002). Gimlet: A computer program for analysing genetic individual identification data. Molecular Ecology Notes, 2, 377–379.

[ece311285-bib-0069] Valière, N. , Fumagalli, L. , Gielly, L. , Miquel, C. , Lequette, B. , Poulle, M.‐L. , Weber, J.‐M. , Arlettaz, R. , & Taberlet, P. (2003). Long‐distance wolf recolonization of France and Switzerland inferred from non‐invasive genetic sampling over a period of 10 years. Animal Conservation, 6, 83–92.

[ece311285-bib-0070] Velli, E. , Fabbri, E. , Galaverni, M. , Mattucci, F. , Mattioli, L. , Molinari, L. , & Caniglia, R. (2019). Ethanol versus swabs: What is a better tool to preserve faecal samples for non‐invasive genetic analyses? Hystrix, 30, 24–29.

[ece311285-bib-0071] Velli, E. , Mattucci, F. , Lazzeri, L. , Fabbri, E. , Pacini, G. , Belardi, I. , Mucci, N. , & Caniglia, R. (2022). “Guess Who's Coming to Dinner”: Molecular tools to reconstruct multilocus genetic profiles from wild canid consumption remains. Animals, 12, 2428. 36139288 10.3390/ani12182428 PMC9495216

[ece311285-bib-0072] Zimen, E. , & Boitani, L. (1975). Number and distribution of wolves in Italy. Zeitschrift für Säugetierkunde, 40, 102–112.

