# Supplementary material for: Estimating distribution and abundance of wide‐ranging species with integrated spatial models: Opportunities revealed by the first wolf assessment in south‐central Italy
Source: Ecol Evol. 2024 May 13;14(5):e11285. doi: 10.1002/ece3.11285 (PMC11091487; doi:10.1002/ece3.11285)
Supplement: Supplementary file 1 — Data S1. [file ECE3-14-e11285-s001.pdf]

Supplemental Information for:

Estimating distribution and abundance of wide-ranging species with integrated population modelling: opportunities revealed by the first wolf assessment in south-central Italy

Gervasi V., Aragno P., Salvatori V., Caniglia R., De Angelis D., Fabbri E., La Morgia V., Marucco F., Velli E., Genovesi P.

Table of Contents:

**Appendix 1** - Description of laboratory methods with details on primers and amplification profiles for all the genotyped markers	Page 2
**Table S1 -** Description of the genotyped autosomal (CFA) and Y-linked (CFAY) microsatellites (STR), Amelogenin and β-defensin CBD103 (K-locus) genes, and the hypervariable part of the mtDNA control-region (mtDNA CR1). M1-6: progressive numbers of the multiplexed amplification reactions.	Page 8
**Table S2 -** Description of the genetic variability obtained analysing the 12-STR genotypes identified from both non-invasively and invasively collected samples.	Page 9
**Appendix 2 –** R Code for the integrated model	Page 11

Appendix 1

Description of laboratory methods with details on primers and amplification profiles for all the genotyped markers

The autosomal and Y-linked STR *loci* were amplified in 6 multiplexed primer mixes (M1, M2, M3, M4, M5, M6) using the Qiagen Multiplex PCR Kit (Qiagen Inc, Hilden, Germany), an ABI GeneAmp® *PCR* System *9700, and the following thermal profile:* 94°C/15 min, 94°C/30 sec, 57°C/90 sec, 72°C/60 sec (30-45 cycles), followed by a final extension step at 72°C for 5 min. Amplifications were carried out in 10 µl total volume, including 2 µl of DNA solution from non-invasively collected samples, or 1 µl of DNA solution from muscle and blood samples, 5 µl Qiagen Multiplex PCR mix, 1 µl Qiagen Q-solution, from 0.1 µl to 0.3 µl of 10 µM primer mix (forward and reverse) and RNase-free water up to the final volume.

The 3-bp deletion (named K^B or $CBD103^{\Delta G23}$) at the *β-defensin CBD103* gene (the *K*-locus) was genotyped as part of the M3, including 0.3 µl of the 10 µM primers CBD103_ΔG23F (TCCGGCACGTTCTGTTTT, 6-FAM) and CBD103_ΔG23R (TTCGGCCAGTGGAAGAAC), while a diagnostic region of the *Amelogenin* gene was amplified as part of the M4, including 0.2 µl of the 10 µM primers AMGF (GTGCCAGCTCAGCAGCCCGTGGT) and AMGR (TCGGAGGCAGAGGTGGCTGTGGC). PCR products were analysed in an ABI 3130XL automated sequencer. The allele sizes of the amplified *loci* were estimated using the ABI ROX-350 size standard and the ABI software GENEMAPPER V.5.0.

The mtDNA control-region was amplified in 10 µl PCR volumes, including 1 µl or 2 µl of DNA solution, 0.3 pmol of the primers WDloopH254 and WDloopL (Caniglia et al. 2013b), using the following thermal profile: 94°C/2 min, 94°C/15 sec, 55°C/15 sec, 72°C/30 sec (40 cycles), followed by a final extension at 72°C for 5 min. PCR products were purified using the exonuclease/shrimp alkaline phosphatase procedure (Exo-Sap; Amersham) and sequenced in both directions using the ABI Big Dye Terminator kit v. 3.1 with the following steps: 96°C/10 sec, 55°C/5 sec, 60°C/4 min of final

extension (25 cycles). PCR products were analysed in an ABI 3130XL automated sequencer using the ABI programs SEQUENCINGANALYSIS v. 3.7 and SEQSCAPE v. 2.5. Detected haplotypes were compared with sequences available from GenBank using Blast (Altschul et al., 1990).

Description of the multiple-tube protocol

The genotypes were identified using a multiple-tube procedure consisting of the following steps. DNA quality was screened by independently amplifying each sample four times at M1 STR *loci* (FH2096, FH2137 and CPH8). Samples showing ≤ 50% positive PCR (PCR+) were directly discarded. Samples showing > 50% PCR+ were amplified four times at the remaining nine autosomal (FH2004, FH2079, FH2088, CPH2, CPH4, CPH5, CPH8, CPH12, C09.250 and C20.253) and at uniparental and coding (Y-linked STRs and K-*locus*; Supplementary Table S1) *loci*. Samples showing a reliability index (R) ≥ 0.950 estimated from the software RELIOTYPE (Miller et al. 2002) were considered reliable at all markers and directly accepted. Unreliable *loci* were further replicated other four times accepting additional samples showing $R \geq 0.950$, and definitively discarding samples showing $R < 0.950$ after eight replicates (Caniglia et al. 2014).

Consensus genotypes were reconstructed from the 4-8 replicated amplifications per *locus* per sample foreseen by the multi-tube approach using the software GIMLET v.1.3.3 (Valière 2002), accepting the heterozygotes only if both alleles were seen in at least two replicates, and the homozygotes only if a single allele was observed in at least four replicates.

GIMLET was also used to match the reconstructed genotypes to each other and with the ISPRA *Canis* database (Caniglia et al. 2020) to identify identical genotypes and individual recaptures.

The consensus genotypes and the 4-8 replicated amplifications per *locus* per sample needed to obtain them were finally used in GIMLET to compute the amplification success (PCR+ = the number of successful PCRs divided by the total number of PCR runs across samples), and estimate the allelic

dropout (ADO = the number of allelic dropouts over the number of successful amplifications of heterozygous genotypes at a given *locus*) and false allele (FA = the number of amplifications leading to one or more false alleles at a *locus* over the total number of successful amplifications at that *locus*) rates (Caniglia et al. 2014).

Taxon identification

The 12-STR *multilocus* genotypes were assigned to their *taxon* of origin (wolf, dog or admixed), independently of any *a prior* non-genetic information, through a Bayesian clustering procedure implemented in the program ParallelStructure (Besnier and Glover 2013), an R package implementing Structure, which estimates individual membership proportions (q_i) to the reference populations and corresponding 90% credibility intervals (CI), speeding up analysis times, and automatically subdividing a dataset of unknown genotypes to be assigned to predefined reference populations into multiple single projects which are independently run, preventing that sample sizes or the simultaneous analysis of samples with different levels of admixture might affect results (Caniglia et al. 2020).

As reference populations for the assignment, we selected 190 unrelated wild individuals belonging to the Italian wolf population (112 males and 78 females, representative of the entire population variability) showing the typical Italian wolf coat colour pattern and neither morphologically nor genetically detectable signs of hybridization (Randi et al. 2014; Galaverni et al. 2017), and 89 wolf-sized dogs (40 males and 49 females) living in rural areas, from the ISPRA *Canis* database (Caniglia et al. 2020). We ran five repetitions of ParallelStructure with 5×10^5 iterations following a burn-in period of 5×10^4 iterations using the *Admixture* (A) and *Independent allele frequencies* (F) models (Falush et al. 2003), and assuming K=2 a *priori* clusters (corresponding to the optimal number of

genetic clusters in which reference populations are split). We used the software CLUMPAK (Kopelman et al. 2015) to concatenate the data from the five independent runs for each *K*.

Based on their assignment membership proportions to the reference wolf population (q_w) and the information derived from the uniparental and functional markers (four Y-linked STRs, K-*locus*), we classified the unknown individual genotypes as: "wolf" if $q_w \geq 0.990$ and no domestic component at the other analysed markers; "recent hybrid" if $q_w < 0.975$; "introgressed wolf" if $0.975 \leq q_w < 0.990$ and/or if they showed a domestic Y-haplotype, as well as the presence of the melanistic deletion at the β-defensin (Caniglia et al. 2020).

Results

Sample genotyping and *Taxon* identification

After the four to eight replicated PCR per sample per *locus* foreseen by the multiple-tube protocol, 971 (61%) of the 1600 non-invasively collected samples were reliable genotyped ($R \geq 0.990$) at the 12 autosomal STRs, showing an average number of positive amplifications per *locus* of 0.90 (ranging from 0.82 to 0.96), and average among *loci* rates of ADO = 0.19 (± 0.04 SD) and FA = 0.0008 (± 0.0010). Regrouping procedures identified them as belonging to 590 individual genotypes (235 females, 312 males and 43 with undetermined gender). All the 32 biological samples obtained from found dead or live-trapped animals produced reliable new genotypes ($R \geq 0.990$) never previously sampled (23 females and 9 males), showing no evidence of ADO or FA errors.

Finally, joining the genotypes detected from the invasively and non-invasively collected samples, a total of 622 individual genotypes were identified and definitely accepted ($R \geq 0.990$): 258 (41%) females, 321 (52%) males and 43 (7%) with undetermined gender. These 622 detected genotypes showed *multilocus* PID = 5.5×10^{-11} and $PID_{sibs} = 6.4 \times 10^{-5}$, meaning that only 6.4 individuals in 100,000 siblings are expected to share by chance an identical genotype, suggesting no ''shadow

effect'' (all the detected genotypes can be considered as distinct individuals; Mills et al. 2000), and that matching genotypes can be considered as recaptures of the same individual. Resampling frequencies were heterogeneous: 423 genotypes (68%) were sampled only once, while the other 199 (31.9%) were sampled from two to 10 times. The resampled individuals also showed highly heterogeneous permanence periods, ranging from a few days to about a few months.

Individual membership proportions to the reference wolf population (q_w) estimated from the Bayesian assignment procedures, according to the selected *q*-thresholds (Caniglia et al. 2020), classified the 622 12-STR individual genotypes as: 395 (183 females, 187 males, 25 with undetermined gender) wolves ($q_w \geq 0.990$), 109 (27 females, 75 males, 7 with undetermined gender) dogs ($q_w < 0.100$), 60 (26 females, 29 males, 5 with undetermined gender) recent wolf-dog hybrids ($q_w < 0.975$) and 58 (22 females, 30 males, 6 undetermined gender) introgressed wolves ($0.975 \leq q_w < 0.990$; Table S2).

When the results obtained from the assignment procedures were integrated with the uniparental (mtDNA CR, four Y-linked STRs) and coding (K-*locus*) data, 22 wolves were reclassified as introgressed individuals since 16 males (corresponding to 9% of the wolf males) showed dog Y-haplotypes and 6 (1.5%; 3 females and 3 males) showed the melanistic 3bp deletion. Additionally, among the 58 introgressed individuals, 3 males also showed a dog Y-haplotype and one female (1.7%) showed the melanistic 3bp deletion. Among the 60 recent wolf-dog hybrids, 8 males (corresponding to 28% of the wolf-dog hybrid males) also showed a dog Y-haplotype and 6 individuals (10%; 3 females, 2 males and 1 with undetermined gender) also the melanistic 3bp deletion. All the individuals identified as wolves, recent hybrids or introgressed animals showed mtDNA haplotypes typical of the Italian wolf population (Montana et al 2017).

Finally, multi-marker genetic analyses identified a total of 513 wild canid genotypes (231 females, 246 males, 36 undetermined gender), corresponding to 373 (180 females, 168 males, 25 with

undetermined gender) wolves, 60 (26 females, 29 males, 5 with undetermined gender) recent wolf-dog hybrids and 80 (25 females, 49 males, 5 with undetermined gender) introgressed wolves, showing PID = 2.8 x 10^{-9} and PID_{sibs} = 2.2 x 10^{-4}, mean number of different alleles N_A = 8.1 (± 0.9 SE), mean number of effective alleles N_E = 2.9 (± 0.3 SE), observed heterozygosity H_O = 0.57 (± 0.05 SE) and expected heterozygosity H_E = 0.60 (± 0.05 SE). These 513 canid *multilocus* genotypes and their resamplings were used as capture-mark-recapture (CMR) records to reconstruct individual encounter histories and carry out the demographic analyses as explained in the next sections.

Table S1. Description of the genotyped autosomal (CFA) and Y-linked (CFAY) microsatellites (STR), *Amelogenin* and ***β-defensin CBD103*** (K-*locus*) genes, and the hypervariable part of the mtDNA control-region (mtDNA CR1). M1-6: progressive numbers of the multiplexed amplification reactions.

Locus	**Chromosome**	**STR repeat size**	**Allele sizes (bp)**	**Dye label**	**Multiplex**	**Reference**
FH2096	CFA11	Tetranucleotide	86-110	FAM	M1	Francisco et al. 1996
FH2137	CFA3	Dinucleotide	140-192	HEX	M1	Francisco et al. 1996
CPH8	CFA13	Dinucleotide	191-219	FAM	M1	Fredholm et al. 1995
FH2004	CFA11	Tetranucleotide	104-202	HEX	M2	Francisco et al. 1996
CPH2	CFA32	Dinucleotide	88-106	FAM	M2	Fredholm et al. 1995
FH2079	CFA24	Tetranucleotide	246-282	HEX	M3	Francisco et al. 1996
FH2088	CFA15	Dinucleotide	91-139	FAM	M3	Francisco et al. 1996
K-*locus*	CFA16	Codon deletion	147-151	FAM	M3	Caniglia et al. 2013a
CPH4	CFA15	Dinucleotide	130-155	FAM	M4	Fredholm et al. 1995
CPH5	CFA15	Dinucleotide	102-124	HEX	M4	Fredholm et al. 1995
Amelogenin	CFAX	-	174-218	FAM	M4	Randi et al. 2014
CPH12	CFA8	Dinucleotide	188-214	HEX	M5	Fredholm et al. 1995
C09.250	CFA9	Dinucleotide	121-145	HEX	M5	Ostrander et al. 1993
C20.253	CFA20	Dinucleotide	90-120	HEX	M5	Ostrander et al. 1993
MSY34A	CFAY	Dinucleotide	160-190	FAM	M6	Sundqvist et al. 2001
MSY41A	CFAY	Dinucleotide	90-150	HEX	M6	Sundqvist et al. 2001
MSY34B	CFAY	Dinucleotide	167-177	HEX	M6	Sundqvist et al. 2001
MSY41B	CFAY	Dinucleotide	109-137	FAM	M6	Sundqvist et al. 2001
mtDNA CR1	mtDNA	-	250	-	-	Caniglia et al. 2013b

Table S2 – Description of the genetic variability obtained analysing the 12-STR genotypes identified from both non-invasively and invasively collected samples.

Taxon (N)	Na	Ne	Ho	He	uHe
Wolves (373)	6.9 (0.8)	2.8 (0.3)	0.55 (0.06)	0.58 (0.06)	0.58 (0.06)
Introgressed wolves (80)	6.0 (0.7)	3.0 (0.3)	0.59 (0.05)	0.61 (0.05)	0.62 (0.05)
Recent wolf-dog admixed individuals (60)	6.5 (0.7)	3.3 (0.3)	0.66 (0.04)	0.66 (0.04)	0.67 (0.04)
Overall (513)	8.1 (0.9)	2.9 (0.3)	0.57 (0.05)	0.60 (0.05)	0.60 (0.05)

N: sample size; Na: number of observed alleles; Ne: number of effective alleles; Ho: observed heterozygosity; He: expected heterozygosity; uHe: unbiased expected heterozygosity. Standard errors are in parentheses.

Supplementary References

Altschul SF, Gish W, Miller W, Myers EW, Lipman DJ Basic local alignment search tool. Journal of Molecular Biology. 1990;215(3):403–410. doi: 10.1016/S0022-2836(05)80360-2.

Besnier, F. & Glover, K. A. Parallel Structure: a R package to distribute parallel runs of the population genetics program Structure on multi-core computers. Plos One 8, e70651 (2013).

Caniglia R, Fabbri E, Greco C, Galaverni M, Manghi L, Boitani L, Sforzi A, Randi E (2013a) Black coats in an admixed wolf × dog pack: is melanism an indicator of hybridization in wolves? European Journal of Wildlife Research 59: 543-555.

Romolo Caniglia, Elena Fabbri, Luigi Mastrogiuseppe, Ettore Randi (2013b) Who is Who? Identification of livestock predators using forensic genetic approaches. Forensic Science International Genetics, 7: 397-404

Caniglia, R., Fabbri, E., Galaverni, M., Milanesi, P. & Randi, E. Noninvasive sampling and genetic variability, pack structure, and dynamics in an expanding wolf population. J. Mammal. 95, 41–59 (2014).

Caniglia R, Galaverni M, Velli E, Mattucci F, Canu A, Apollonio M, Mucci N, Scandura M and Fabbri E (2020) A standardized approach to empirically define reliable assignment thresholds and appropriate management categories in deeply introgressed populations. Sci Rep 10, 2862. DOI: https://doi.org/10.1038/s41598-020-59521-2

Falush D., Stephens M. & Pritchard J. K. Inference of population structure using multilocus genotype data: linked loci and correlated allele frequencies. Genetics 164, 1567–1587 (2003).

Francisco L, Langsten A, Mellersh C, Neal C, Ostrander E (1996) A class of highly polymorphic tetranucleotide repeats for canine genetic mapping. Mammalian Genome 7: 359-362.

Fredholm M, Winterø A (1995) Variation of short tandem repeats within and between species belonging to the Canidae family. Mammalian Genome 6: 11-18.

Galaverni M, Caniglia R, Pagani L, Fabbri E, Boattini A and Randi E. Disentangling timing of admixture, patterns of introgression and phenotypic indicators in a hybridizing wolf population. Mol. Biol. Evol. 34, 2324–2339 (2017).

Kopelman NM, Mayzel J, Jakobsson M, Rosenberg NA, Mayrose I (2015) Clumpak: a program for identifying clustering modes and packaging population structure inferences across K. Mol. Ecol. Resour. 15(5):1179-1191.

Miller C.R., Joyce P., Waits L.P., 2002. Assessing Allelic Dropout and Genotype Reliability Using Maximum Likelihood. Genetics 160: 357 LP-366.

Montana, L. et al. Combining phylogenetic and demographic inferences to assess the origin of the genetic diversity in an isolated wolf population. PLoS One 12, e0176560 (2017).

Ostrander EA, Sprague GF, Rine J (1993) Identification and characterization of dinucleotide repeat (CA) markers for genetic mapping in dog. Genomics 16: 207-213.

Randi, E. et al. Multilocus detection of wolf × dog hybridization in Italy, and guidelines for marker selection. PLoS One 9, e86409 (2014).

Sundqvist AK, Ellegren H, Olivier M, Vila C (2001) Y chromosome haplotyping in Scandinavian wolves (*Canis lupus*) based on microsatellite markers. Molecular Ecology 10: 1959-1966.

Valière, N. Gimlet: a computer program for analysing genetic individual identification data. Mol. Ecol. Notes 2, 377–379, https://doi. org/10.1046/j.1471-8286.2002.00228.x-i2 (2002).

Appendix 2 – Code for the integrated model.

```
code <- nimbleCode({

###################
# SECR MODEL      #
###################

  CRro[1:n.cells] ~ dcar_normal(adj=adj[1:L], num=num[1:n.cells], tau=CRtau)
  probs[1:n.cells] <- CRmu[1:n.cells]/EN

 # density at each cell
     for (k in 1:n.cells){
          CRmu[k] <- exp(CRmu0 + CRmu1 * altitude[k]  + CRmu2 * human[k] +  CRmu3 * forest[k]
+  CRmu4 * ruggedness[k] +  CRmu5 * agriculture[k] +   CRmu6 * agrinat[k] + CRmu7 * roads[k] +
          CRmu8 * urban[k] +  CRmu9 * prey[k] +  CRmu10 * strato_a[k] +  CRmu11 * strato_b[k]
+  CRmu12 * strato_c[k] + CRro[k]) * (1-equals(truocc[k],0)) + const * equals(truocc[k],0)
                 }

          EN <- sum(CRmu[1:n.cells])
          CRpsi <- EN / n.individuals



 # capture probability
for (i in 1:14){
                 CRalpha0[i] ~ dnorm(0, CRtau.alpha0)
                 }
```

```
for (i in 1:n.individuals){
        CRw[i] ~ dbern(CRteta)
        logit(CRp0[i,1:n.detectors]) <- CRalpha0[area[i]] + CRalpha1 * effort[1:n.detectors] +
CRalpha2 * sexF[i] + CRalpha3 * snow[1:n.detectors] + CRw[i] * CRalpha4
    }


 # sigma


    for (k in 1:n.cells){
                # expected number of wolves in each cell
            log(CRsigma[k]) <- CRbeta0 + CRbeta1 * prey[k]
            }




 ## loop over individuals
 for(i in 1:n.individuals) {
  ## AC coordinates
  ac[i]  ~ dunif(0.5, n.cells+0.5)
  nll[i] <- - log(probs[round(ac[i])])
  zeros[i] ~ dpois(nll[i])
  sxy[i,1] ~ dunif((sites[round(ac[i]),1]-0.5), (sites[round(ac[i]),1]+0.5))
  sxy[i,2] ~ dunif((sites[round(ac[i]),2]-0.5), (sites[round(ac[i]),2]+0.5))




  ## habitat constraint
  ones[i] ~ dHabitatMask( s = sxy[i,1:2],
```

```
                    xmin = lowerCoords[1],
                    xmax = upperCoords[1],
                    ymin = lowerCoords[2],
                    ymax = upperCoords[2],
                    habitat = habitat.mx[1:y.max,1:x.max])
  ## latent dead/alive indicators
  z[i] ~ dbern(CRpsi)
  ## likelihood
  y[i, 1:nMaxDetectors] ~ dbinomLocal_normal( detNums = nbDetections[i],
                                  detIndices = yDets[i,1:nMaxDetectors],
                                  size = trials[1:n.detectors],
                                  p0Traps = CRp0[i,1:n.detectors],
                                  s = sxy[i,1:2],
                                  sigma = CRsigma[round(ac[i])],
                                  trapCoords = detector.xy[1:n.detectors,1:2],
                                  localTrapsIndices = DetectorIndex[1:n.cells,1:maxNBDets],
                                  localTrapsNum = nDetectors[1:n.cells],
                                  resizeFactor = ResizeFactor,
                                  habitatGrid = habitatIDDet[1:y.maxDet,1:x.maxDet],
                                  indicator = z[i])
 }
 ## derived quantity: total population size
 N <- sum(z[1:n.individuals])


########################
# OCCUPANCY MODEL      #
########################
```

```
# OBSERVATIONS (+1)
# 0 = non-detected
# 1 = detected as ascertained wolf
# 2 = detected as ascertained dog
# 3 = detected as unknown

# STATES
# 1 = occupied by wolves (W)
# 0 = not occupied (N)

for (k in 1:14){
            chi[k] ~ dnorm(0, tau.chi)
            }



   ro[1:n.cells] ~ dcar_normal(adj=adj[1:L], num=num[1:n.cells], tau=tau)
   nu[1:n.cells] ~ dcar_normal(adj=adj[1:L], num=num[1:n.cells], tau=tau2)

  for (i in 1:n.cells){

  logit(m1[i]) <- alpha0  + alphaEffort*T_effort_st[i] + alphaUrb * urban[i] + alphaRoadL * roads[i]
+ alphaHuman * human[i]  + ro[i]

  logit(G[i]) <-  chi[areas[i]]

  g[i] <- G[i]*lab[i]

      truocc[i] ~ dbern(psi[i]) # true occupancy for site i
```

```
    logit(psi[i]) <- (beta0  + betaForest * forest[i] + betaAlt * altitude[i] + betaUrb * urban[i] +
betaRoadL * roads[i] +
    betaAgric * agriculture[i]  + betaAgrinat * agrinat[i] + betaHuman * human[i] + betaRugg *
ruggedness[i] + betaPrey * prey[i]  + nu[i])
    q[i] ~ dbern(het_p)

        for (j in 1:K) {

     p[i,j,1,1] <- (1-p1[i,j])
     p[i,j,1,2] <- 0
     p[i,j,1,3] <- p1[i,j]*g[i]
     p[i,j,1,4] <- p1[i,j]*(1-g[i])
     p[i,j,2,1] <- 1-p2[i,j]
     p[i,j,2,2] <- p2[i,j]*g[i]*(1-m1[i])
     p[i,j,2,3] <- p2[i,j]*g[i]*m1[i]
     p[i,j,2,4] <- p2[i,j]*(1-g[i])

          logit(p1[i,j]) <- delta0   + deltaEffort * effort_st[i,j] + deltaBimestre1 * Bim1[j] +
deltaBimestre2 * Bim2[j] + deltaBimestre3 * Bim3[j]  + deltaSnow * snow[i] +
                                 q[i] * deltahetH + (1-q[i]) * deltahetL

      logit(p2[i,j]) <- gamma0   + gammaEffort * effort_st[i,j] + gammaBimestre1 * Bim1[j] +
gammaBimestre2 * Bim2[j] + gammaBimestre3 * Bim3[j] + gammaSnow * snow[i] +
                                 q[i] * gammahetH + (1-q[i]) * gammahetL

      mydata[i,j] ~ dcat(p[i,j,(truocc[i]+1),1:4])
        }
```

```
  }


# PHOTO-TRAPS

for (i in 1:n.cells){

      b[i] ~ dbern(het_p.photo)

            for (j in 1:K) {

       p.f[i,j,1,1] <- 1
       p.f[i,j,1,2] <- 0
       p.f[i,j,1,3] <- 0
       p.f[i,j,1,4] <- 0
       p.f[i,j,2,1] <- 1 - p.photo[i,j]
       p.f[i,j,2,2] <- p.photo[i,j]
       p.f[i,j,2,3] <- 0
       p.f[i,j,2,4] <- 0


        logit(p.photo[i,j]) <- epsilon0 + epsilonEffort * photo_effort_st[i,j] + epsilonBimestre1 *
Bim1[j] + epsilonBimestre2 * Bim2[j] + epsilonBimestre3 * Bim3[j] +
                                                    b[i] * epsilonhetH + (1-b[i]) * epsilonhetL


        mydata.photo[i,j] ~ dcat(p.f[i,j,(truocc[i]+1),1:4])
            }
            }
```

```
## SECR priors

  #density
  CRmu0 ~  dnorm(0, 1)
  CRmu1 ~  dnorm(0, 1)
  CRmu2 ~ dnorm(0, 1)
  CRmu3 ~  dnorm(0, 1)
  CRmu4 ~  dnorm(0, 1)
  CRmu5 ~  dnorm(0, 1)
  CRmu6 ~  dnorm(0, 1)
  CRmu7 ~  dnorm(0, 1)
  CRmu8 ~  dnorm(0, 1)
  CRmu9 ~  dnorm(0, 1)
  CRmu10 ~  dnorm(0, 1)
  CRmu11 ~  dnorm(0, 1)
  CRmu12 ~  dnorm(0, 1)
  CRtau <- 1 / CRsigma2.tau
  CRsigma2.tau ~ dunif(0,200)

  # detection
  CRalpha1 ~  dnorm(0, 1)
  CRalpha2 ~  dnorm(0, 1)
  CRalpha3 ~  dnorm(0, 1)
  CRalpha4 ~  dnorm(0, 1)
  CRalpha5 ~  dnorm(0, 1)
  CRteta ~  dunif(0, 1)
  CRtau.alpha0 <- 1 / CRsigma2.alpha0
```

```
  CRsigma2.alpha0 ~ dunif(0,200)

  # sigma
  CRbeta0 ~  dnorm(0, 1)
  CRbeta1 ~  dnorm(0, 1)
  CRtau.chi <- 1 / CRsigma2.tau.chi
  CRsigma2.tau.chi ~ dunif(0,200)


# OCCUPANCY priors

 #m1
  alpha0 ~  dnorm(0,5)
  alphaEffort ~  dnorm(0,5)
  alphaUrb ~  dnorm(0,5)
  alphaRoadL ~  dnorm(0,5)
  alphaHuman ~ dnorm(0,5)
  tau <- 1 / sigma2.tau
  sigma2.tau ~ dunif(0,200)

  #G
  tau.chi <- 1 / sigma2.chi
  sigma2.chi ~ dunif(0,200)

  # psi
  beta0 ~  dnorm(0,5)
  betaForest ~  dnorm(0,5)
  betaAlt ~  dnorm(0,5)
  betaUrb ~  dnorm(0,5)
```

```
betaRoadL ~  dnorm(0,5)
betaAgric ~ dnorm(0,5)
betaAgrinat ~ dnorm(0,5)
betaHuman ~ dnorm(0,5)
betaRugg ~ dnorm(0,5)
betaPrey ~ dnorm(0,5)
tau2 <- 1 / sigma2.tau2
sigma2.tau2 ~ dunif(0,200)

# p1
delta0 ~ dnorm(0,5)
deltaEffort ~ dnorm(0,5)
deltaBimestre1 ~ dnorm(0,5)
deltaBimestre2 ~ dnorm(0,5)
deltaBimestre3 ~ dnorm(0,5)
deltaSnow ~ dnorm(0,5)
deltahetH  ~ dnorm(0,5)
deltahetL  ~ dnorm(0,5)
het_p ~ dunif(0,1)


# p2
gamma0 ~ dnorm(0,5)
gammaEffort ~ dnorm(0,5)
gammaBimestre1 ~ dnorm(0,5)
gammaBimestre2 ~ dnorm(0,5)
gammaBimestre3 ~ dnorm(0,5)
gammaSnow ~ dnorm(0,5)
gammahetH  ~ dnorm(0,5)
```

```
  gammahetL  ~ dnorm(0,5)

# p.photo
  epsilon0 ~ dnorm(0,5)
  epsilonEffort ~ dnorm(0,5)
  epsilonBimestre1 ~ dnorm(0,5)
  epsilonBimestre2 ~ dnorm(0,5)
  epsilonBimestre3 ~ dnorm(0,5)
  epsilonhetH  ~ dnorm(0,5)
  epsilonhetL  ~ dnorm(0,5)
  het_p.photo ~ dunif(0,1)


})




# INITIAL VALUES

inits <- list(
# SECR
sxy = wolf.inits$sxy,
            z = wolf.inits$z,
                        ac = my.wolf.input$ac,

  #density

                        CRmu0 = 0,
```

```
            CRmu1 = 0,
            CRmu2 = 0,
            CRmu3 = 0,
            CRmu4 = 0,
            CRmu5 = 0,
            CRmu6 = 0,
            CRmu7 = 0,
            CRmu8 = 0,
            CRmu9 = 0,
            CRmu10 = 0,
            CRmu11 = 0,
            CRmu12 = 0,
            CRsigma2.tau = 10,
CRro = rep(0,constants$n.cells),

            # detection
            CRalpha1 = 0,
            CRalpha2 = 0,
            CRalpha3 = 0,
            CRalpha4 = 0,
            CRalpha5 = 0,
            CRalpha0 = rep(0,14),
            CRsigma2.alpha0 = 10,
CRchi = rep(0,constants$n.cells),
            CRsigma2.tau.chi = 10,
            CRteta = 0.5,
            CRp0 = matrix(0, nrow=constants$n.individuals, ncol=constants$n.cells),
            CRw = rep(0, constants$n.individuals),
```

```
                    #sigma
                    CRbeta0 = 0,
                    CRbeta1 = 0,
          CRchi = rep(0,constants$n.cells),

#OCCUPANCY
#m1
alpha0 = 0.5,
alphaEffort = 0.5,
alphaUrb = 0,
alphaRoadL = 0,
alphaHuman = 0,
sigma2.tau = 10,
ro = rep(0,constants$n.cells),

#G
sigma2.chi = 10,
chi = rep(0,14),

#psi
beta0 = 0,
betaForest = 0,
betaAlt = 0,
betaUrb = 0,
betaRoadL = 0,
betaAgric = 0,
betaAgrinat = 0,
betaHuman = 0,
betaRugg = 0,
```

```
betaPrey = 0,
sigma2.tau2 = 10,
nu = rep(0,constants$n.cells),

#p1
delta0 = 0,
deltaEffort = 0,
deltaBimestre1 = 0,
deltaBimestre2 = 0,
deltaBimestre3 = 0,
deltaSnow = 0,
deltahetH = 0,
deltahetL = 0,
het_p = 0.5,
q = rep(0,constants$n.cells),

#p2
gamma0 = 0,
gammaEffort = 0,
gammaBimestre1 = 0,
gammaBimestre2 = 0,
gammaBimestre3 = 0,
gammaSnow = 0,
gammahetH = 0,
gammahetL = 0,

#p.photo
epsilon0 = 0,
epsilonEffort = 0,
```

```
epsilonBimestre1 = 0,
epsilonBimestre2 = 0,
epsilonBimestre3 = 0,
epsilonhetH = 0,
epsilonhetL = 0,
het_p.photo = 0.5,
b = rep(0,constants$n.cells),
truocc = wolf.inits$truocc_inits)
```
